# Sp100 colocalizes with HPV replication foci and restricts the productive stage of the infectious cycle

**DOI:** 10.1371/journal.ppat.1006660

**Published:** 2017-10-02

**Authors:** Wesley H. Stepp, James D. Stamos, Simran Khurana, Alix Warburton, Alison A. McBride

**Affiliations:** Laboratory of Viral Diseases, National Institute of Allergy and Infectious Diseases, National Institutes of Health, Bethesda, Maryland, United States of America; University of Wisconsin Madison School of Medicine and Public Health, UNITED STATES

## Abstract

We have shown previously that Sp100 (a component of the ND10 nuclear body) represses transcription, replication and establishment of incoming human papillomavirus (HPV) DNA in the early stages of infection. In this follow up study, we show that Sp100 does not substantially regulate viral infection in the maintenance phase, however at late stages of infection Sp100 interacts with amplifying viral genomes to repress viral processes. We find that Sp100 localizes to HPV16 replication foci generated in primary keratinocytes, to HPV31 replication foci that form in differentiated cells, and to HPV16 replication foci in CIN 1 cervical biopsies. To analyze this further, Sp100 was down regulated by siRNA treatment of differentiating HPV31 containing cells and levels of viral transcription and replication were assessed. This revealed that Sp100 represses viral transcription and replication in differentiated cells. Analysis of Sp100 binding to viral chromatin showed that Sp100 bound across the viral genome, and that binding increased at late stages of infection. Therefore, Sp100 represses the HPV life cycle at both early and late stages of infection.

## Author summary

Host restriction factors act to limit viral infection as part of the intrinsic immune system. Upon entry into cells, human papillomaviruses (HPV) encounter anti-viral nuclear bodies, called ND10, and components of these bodies, such as Sp100, restrict early viral infection. If HPV escapes these defenses, it can establish a long-term, persistent infection. The eventual production of infectious HPV particles depends on the differentiation program of host keratinocytes. Here we show that in the transition from persistent to productive infection in differentiated keratinocytes, HPV again engages the Sp100 component of ND10 bodies. Sp100 is observed surrounding and inside replication centers in differentiated cells in culture, and in HPV-infected cervical lesions. This results in restriction of viral DNA amplification and late viral gene expression. These data collectively show that Sp100 mediates an anti-viral response at both early and late stages of viral infection.

## Introduction

Human papillomaviruses (HPVs) establish a persistent infection in the cutaneous and mucosal epithelia of their hosts [1]. The virus infects the basal layer of keratinocytes through a micro-fissure and establishes a persistent reservoir of infection in these dividing cells. When the infected cells differentiate during the process of tissue renewal, late viral replication and transcription are induced, and viral particles assemble in the most superficial layers of the epithelium. This persistent, differentiation-dependent life cycle requires several different stages of viral DNA replication: immediately upon infection there is a limited amplification of viral DNA; next the viral genome must become “established” in the cell and be maintained at a low copy number as an extrachromosomal replicon for many cell divisions; and finally, the viral genome must amplify to very high levels in differentiated cells [2].

Like many other DNA viruses, the early stages of HPV transcription and replication initiate at, or adjacent to, the nuclear structure, ND10 [3]. During primary infection, the viral minor capsid protein, L2, delivers the viral DNA to the ND10 body by interaction with the PML protein, and this is important for efficient infection [4, 5]. Furthermore, L2 causes reorganization of ND10 and the displacement of the ND10 factor, Sp100 [6]. In support of this finding, we have shown previously that Sp100 represses transcription of incoming HPV18 genomes [7].

During the maintenance stage of infection, levels of viral transcription and replication are not dramatically affected by the Sp100 proteins [7, 8]. In cells containing extrachromosomal HPV18 genomes, we find that downregulation of Sp100 increased viral replication and transcription only ~1.5-fold (this was not of statistical significance). Habiger et al. observed a similar increase in HPV31 transcription and replication in CIN612-9E cells, which did reach significance. Furthermore, they showed that interferon (IFN) κ induces Sp100, which in turn represses HPV31 transcription [8].

During the productive stage of the HPV lifecycle, amplification of viral DNA is coincident with epithelial differentiation [9]. This amplification event is marked by a shift in transcriptional initiation from the early to the late promoter [10]. This results in three classes of transcripts: early transcripts that utilize the early promoter and early polyadenylation site; intermediate transcripts that use the late promoter and early polyadenylation site; and late transcripts that use both the late promoter and polyadenylation site [11]. Intermediate transcripts encode E1, E2 and E4 proteins, and late transcripts encode the capsid proteins, L1 and L2. The switch between HPV early and late transcription is highly dependent on host cell differentiation, and viral DNA replication is necessary for maximal late transcription [12].

Here we examine the role of Sp100 on viral genome amplification and viral transcription during the productive stage of the viral lifecycle. We observed that Sp100 associates with replication factories formed by expression of HPV16 E1 and E2 in keratinocytes, as well as replication foci formed upon differentiation in the HPV31 containing cervical cell line CIN612-9E. Sp100 is also associated with HPV replication foci at the onset of DNA amplification in the upper layers of a cervical CIN 1 lesion. We observed that Sp100 primarily repressed late HPV31 mRNA transcription, and limited viral replication, in differentiating cells. Using chromatin immunoprecipitation, we show that Sp100 binds across the viral genome and that binding increases upon differentiation. Together, these data show that Sp100 functions as a host restriction factor at both early and late times of the HPV lifecycle.

## Results

### E1/E2 replication compartments localize with the ND10 components PML and Sp100

**Fig 1:**
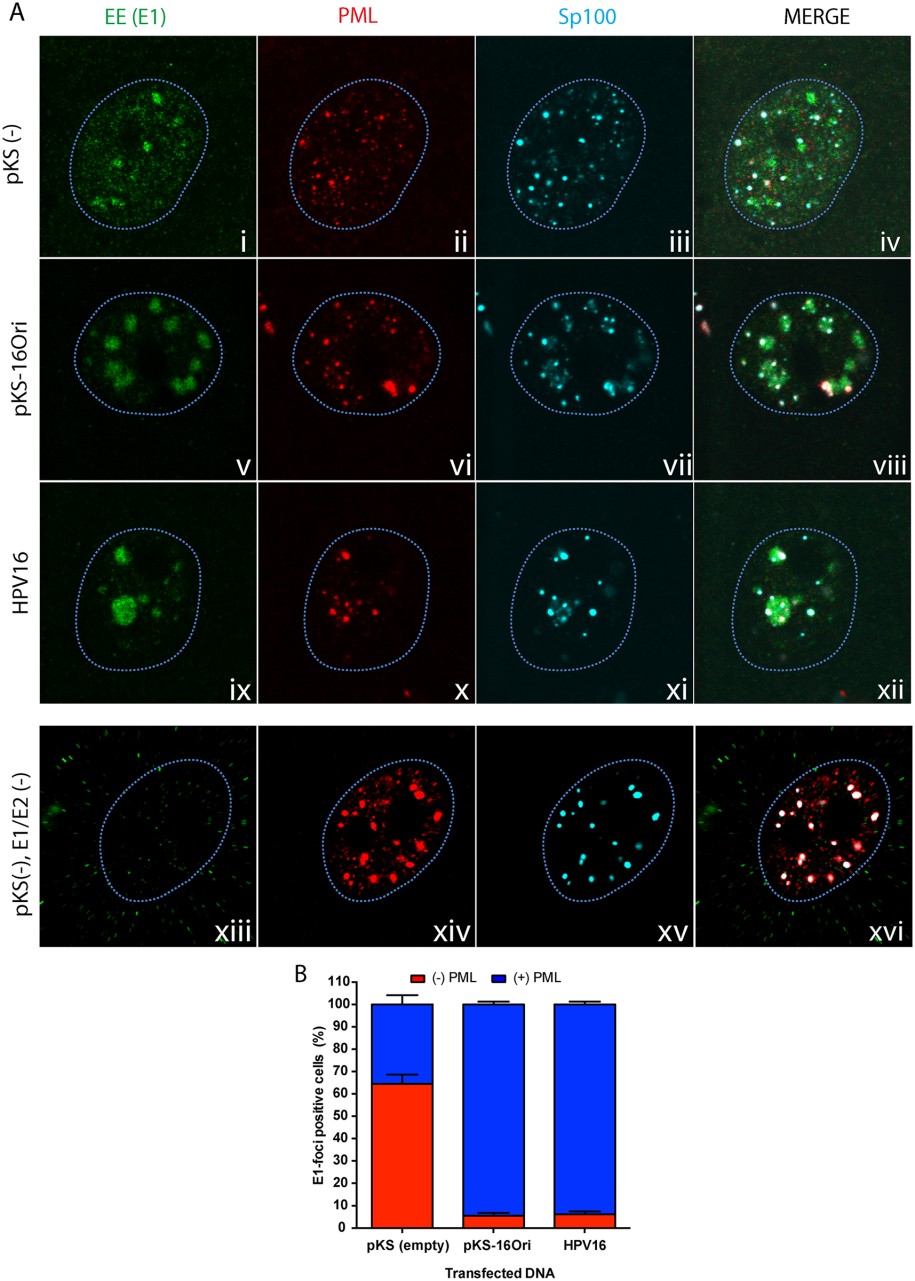
PML and Sp100 association with HPV16 E1/E2 replication foci increases in the presence of replicating viral DNA. **A.** HFK 1A cells cultured on glass coverslips were transfected with HPV16 E1 and E2 expression vectors (400 ng each) and 50 ng of pKS (empty vector), pKS-16Ori, or recircularized HPV16 genome. E1 and E2 expression was induced 24 hours post-transfection with 3 μM CdSO_4_ for four hours and cells were fixed with 4% PFA for analysis by indirect immunofluorescence. Cells were stained with EE (E1, green), PML (red) or Sp100 (cyan) antibodies. Nuclei (outlined in light blue) were detected with DAPI counterstain. Images are single optical slices collected by confocal microscopy, and are representative of three independent transfections. B. E1-foci positive cells were scored for association with PML by visually assessing PML’s proximity to the E1-foci. Results were calculated from a minimum of 20 cells per experiment derived from three independent experiments (Total N = 60). Error bars represent +/-SEM.

Nuclear foci that replicate HPV DNA can be formed by coexpression of the viral E1 and E2 proteins, and these have been previously shown to localize adjacent to ND10 bodies [3]. To investigate this further and to determine if Sp100 is also recruited to sites of viral DNA replication, we generated HPV16 replication foci in a human foreskin keratinocyte (HFK) conditionally immortalized cell line, 1A, as previously described [13]. 1A cells were transfected with HPV16-E1 (pMEP9-16 EE-E1) and HPV16-E2 (pMEP4-16 FLAG-E2) expression vectors, along with either empty vector DNA (pKS), HPV16 origin-containing DNA (pKS-16Ori) or the recircularized HPV16 viral genome. The localization of the viral replication proteins with respect to PML and Sp100 was assessed by confocal microscopy. Both PML and Sp100 localized in nuclear foci (ND10 bodies), which were often observed in association with the E1/E2 foci. In the absence of a viral replicon, PML localized with E1/E2 foci in ~30–40% cells (Fig 1A, panels i-iv and Fig 1B). Introduction of replication competent DNA (pKS-16Ori or a recircularized HPV16 genome) increased the association of PML with E1/E2 foci to >95% (Fig 1A, v-viii and ix-xii; Fig 1B). This confirmed that the association of PML with the HPV replication proteins is enhanced in the presence of replicating viral DNA [3], and showed that Sp100 is also associated with HPV replication foci.

**Fig 2:**
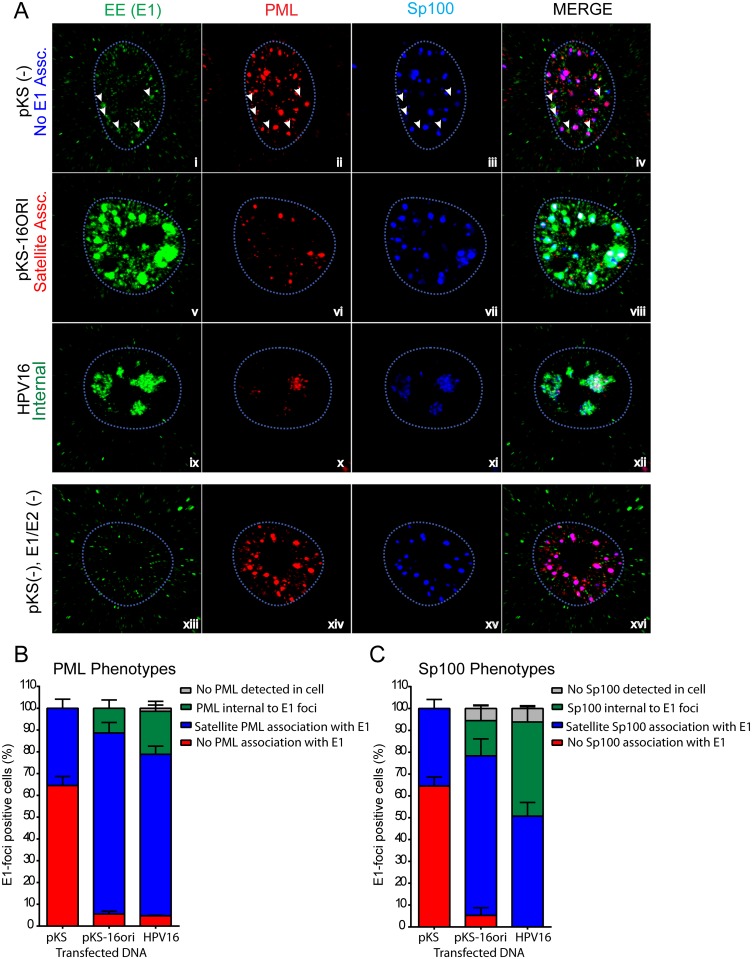
PML and Sp100 associate with HPV replication factories. A. Using the same data as Fig 1, cells were assessed for the predominant phenotypes observed for PML and Sp100 association with replication foci. Images are single optical slices collected by confocal microscopy and representative of three independent transfections. The tip of each arrow in panels i-iv indicates the location of E1/E2 foci. B and C. E1-foci positive cells were scored for four different PML (C) or Sp100 (D) phenotypes: No PML/Sp100 (grey), No E1 association (blue), satellite association (red) or internal to replication foci (green). Graph comprised of data from a minimum of 20 cells from three independent experiments (Total N = 60). Error bars represent +/-SEM.

PML and Sp100 associated with HPV replication foci in either a satellite association around or adjacent to E1/E2 foci, or internally within the replication factories. This internal association was seen only in the presence of replicating viral DNA and was observed more frequently in the presence of the entire HPV16 genome than the plasmid containing only an HPV16 origin (Fig 2A, 2B and 2C).

### Sp100 is observed inside viral replication factories in close association with viral DNA

**Fig 3:**
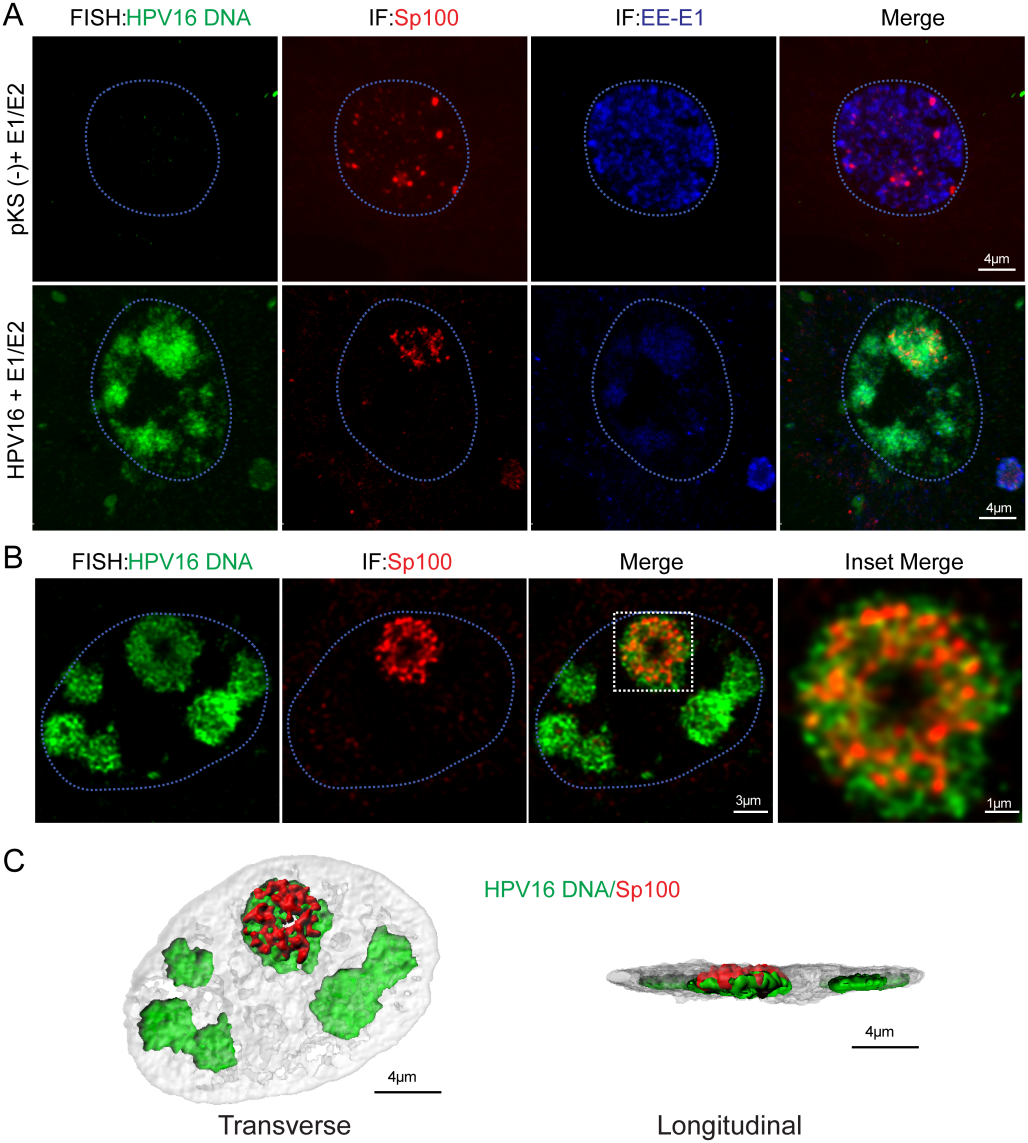
Sp100 is found inside viral replication factories in close association with viral DNA. 1A cells cultured on glass coverslips were transfected with HPV16 E1 and E2 expression vector (400 ng each) and 50 ng recircularized HPV16 genome. Cells were induced 24 hours after DNA transfection with 3 μM CdSO_4_ for four hours and fixed with 4% PFA for confocal microscopy analysis. A. Combined FISH-IF for HPV16 DNA (green), Sp100 protein (red) and EE-E1 (blue). Nuclei (outlined in light blue) were detected with DAPI counterstain. 35 cells were imaged and figure is representative of a single FISH-IF experiment. Images were obtained from a single optical slice. B. Combined FISH-IF for HPV16 DNA (green) and Sp100 protein (red). Nuclei (outlined in light blue) were detected with DAPI counterstain. Magnification of boxed area demonstrates Sp100 (red) association with viral DNA (green). Images shown are single, deconvolved slices obtained from z-stacks collected throughout the nucleus in 0.13 μm per slice. C. 3D reconstruction of panel B showing viral DNA and Sp100 in proximity to each other inside the replication factory. Surface-rendering was generated in IMARIS from a z-stack image (0.13 μm slices) collected at optimum X, Y and Z settings and deconvolved. Images for B and C are representative of three independent experiments with a minimum of 20 cells per experiment analyzed.

To better assess whether increased internal association of Sp100 in viral replication factories inhibited viral DNA synthesis, we directly detected HPV16 DNA in the foci by FISH. Similar to the data presented in Fig 2, Sp100 was arranged in either a satellite configuration around viral DNA replication factories in cells stained for Sp100 and HPV16 DNA, or was found completely inside the entire replication factory (Fig 3A). Further, our analysis of Sp100 localization, in conjunction with 3D image processing, showed that internal Sp100 was closely associated, rather than colocalized with viral DNA in replication foci (Fig 3B and 3C). Notably, most cells that exhibited this internal Sp100 phenotype contained all available Sp100 in a single, and often largest, replication factory (Fig 3B). However, it was difficult to conclude whether Sp100 was repressing viral DNA replication in these factories due to the substantial amounts of viral DNA in these foci.

### PML and Sp100 associate with viral DNA factories in CIN612-9E cells

**Fig 4:**
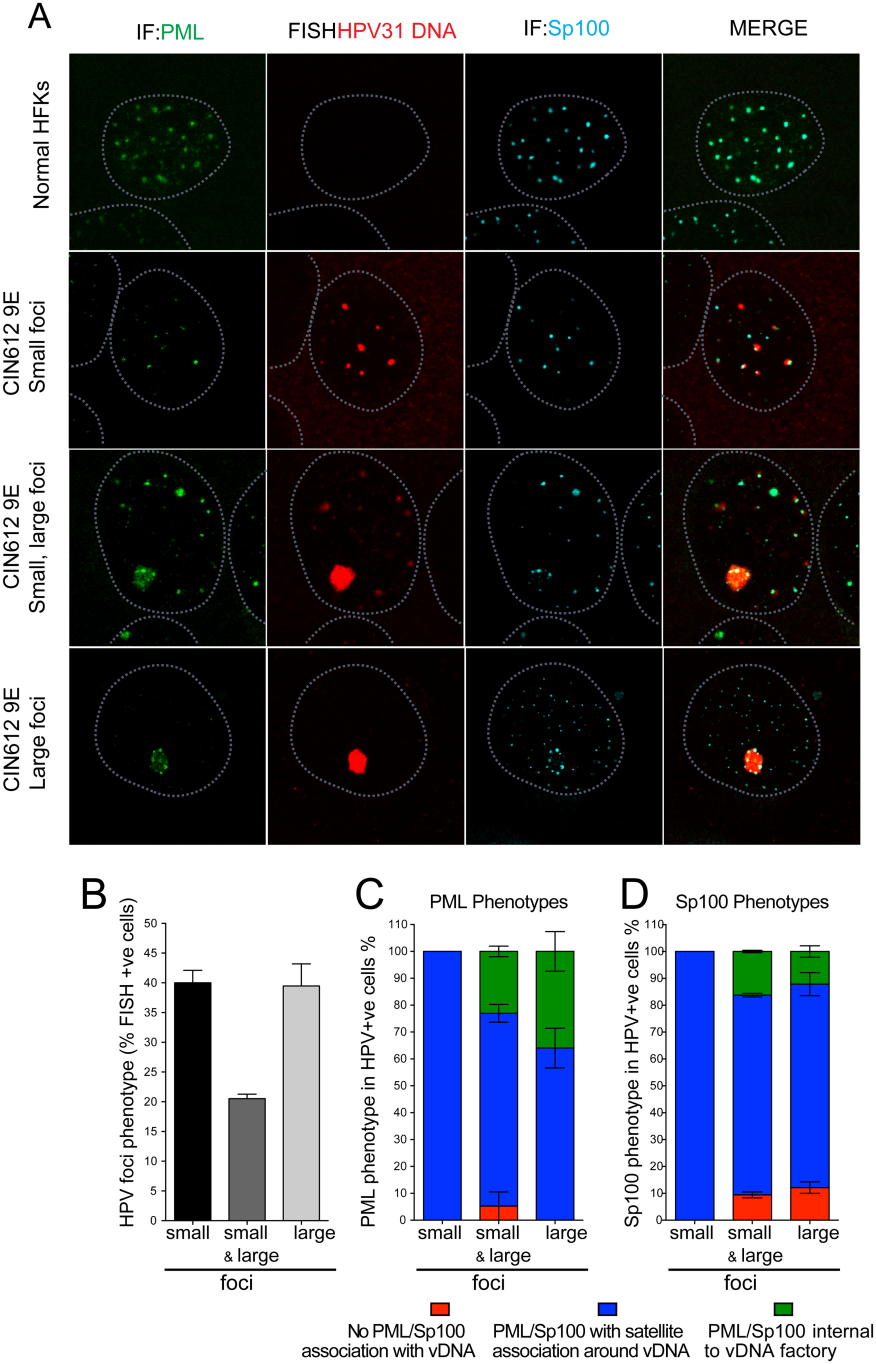
ND10 proteins PML and Sp100 associate with viral DNA foci in CIN612-9E cells. HFKs or CIN612-9E cells were grown on glass coverslips and differentiated for five days with CaCl_2_ and analyzed by FISH-IF. Cells are representative images and quantitation represents at least 57 cells counted from two independent FISH-IF experiments (Total N>100 cells). **A.** FISH-IF for HPV31 DNA (Alexa 555, red), PML (488 green) and Sp100 proteins (647 cyan). Nuclei (outlined in light blue) were detected with DAPI. **B.** Distribution of small and large HPV31 positive replication foci in differentiated CIN612-9E cells. **C.** Distribution of PML association with small and large replication foci in CIN612-9E differentiated cells. **D.** Distribution of Sp100 association with small and large replication foci in CIN612-9E differentiated cells.

To further analyze the association of PML and Sp100 with replication foci, we examined the cell line, CIN612-9E, which is derived from an HPV31-positive cervical biopsy [14] and is frequently used to study the later stages of the HPV lifecycle [9]. 9E cells were grown on glass coverslips to confluence and cultured for five days in high calcium medium to induce differentiation. This results in amplification of viral DNA in nuclear foci when detected by HPV31 FISH. In CIN612-9E cells, we observed three replication foci phenotypes: cells containing several small nuclear foci of HPV31 DNA; cells with a single large focus of viral DNA; or cells containing a mixture of the two (Fig 4A and 4B).

Following differentiation, cells were fixed and combined FISH-IF was performed for HPV31 DNA and the PML and Sp100 proteins. In cells showing multiple, small, punctate FISH signals, almost 100% HPV31 DNA was either partially colocalized or adjacent to both PML and Sp100 nuclear foci (satellite association) (Fig 4C). Furthermore, PML and Sp100 staining patterns were nearly identical, indicating a high degree of colocalization between the two proteins. However, in cells with a singular large focus of viral DNA, Sp100 and PML were observed inside the replication factory in ~15% or ~35% cells, respectively, and displayed a satellite association in most of the remaining cells (Fig 4C). When Sp100 was found inside viral replication factories, PML was sometimes retained outside the foci or sometimes internally along with Sp100. In ~10% cells, large replication foci were devoid of any Sp100 staining but remained associated with PML. We speculate that Sp100 may have been degraded within these large factories to overcome the anti-viral, repressive effects of Sp100.

### Sp100 does not repress viral replication, or transcription in the maintenance phase of infection

**Fig 5:**
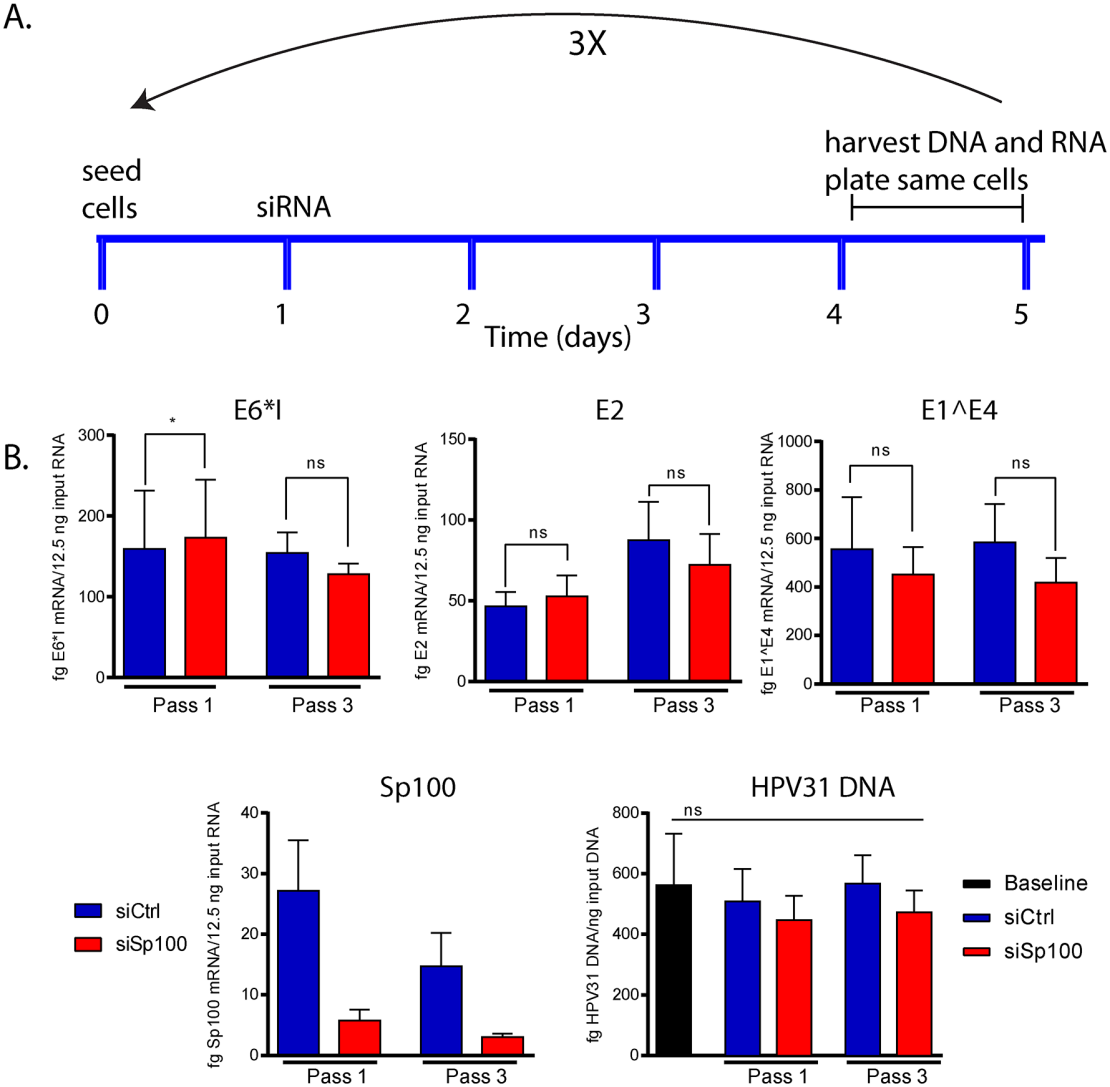
Sp100 has no effect on transcription and replication of HPV31 genomes that are stably maintained in CIN612-9E cells. **A.** Timeline of experiment. Cells were plated at low density and treated with siCtrl or siSp100 RNA after 24 hours. RNA and DNA was harvested on day 4 or 5 (before cells became confluent) and cells were replated for another round of siRNA knockdown. This treatment was continued for three passes. **B.** qPCR was used to measure viral transcription and viral DNA replication after long-term knockdown of Sp100. The efficiency of knockdown was also monitored by measuring levels of Sp100 transcripts. Statistical significance was determined using a paired t-test for transcript abundance and one-way ANOVA to analyze long-term changes in HPV replication. *, P<0.05; ns, not statistically significant. Error bars represent +/- SEM from three independent experiments.

Using CIN612-9E cells, we can study the regulation of the maintenance and the productive stages of the viral life cycle by directly measuring viral transcription and replication. To address the role of Sp100 in the maintenance phase, we assessed the effect of siRNA depletion of Sp100 on early and intermediate HPV mRNAs, and on viral genome copy number over three cell passes. 9E cells were seeded at low density and treated with either Ctrl (control) or Sp100 siRNA 24 hours later. Cells were harvested between 72 hours and 96 hours later and replated at low density. This process was repeated two more times and cellular DNA and RNA were collected at the end of pass 1 and pass 3 (Fig 5A). Levels of E6*I, E1^E4 and E2 viral transcripts and Sp100 depletion efficiency were monitored by qPCR (Fig 5B). This showed efficient downregulation of Sp100 over the three passes, but there was no consistent increase or decrease in viral mRNA after this treatment. Correspondingly, we were unable to observe reproducible changes in viral DNA copy number after three passes of Sp100 downregulation. Similarly, we concluded previously that Sp100 had only minimal effect on the levels of viral RNA and DNA in a cell line containing extrachromosomally replicating HPV18 [7]. Therefore, Sp100 does not affect viral transcription or replication in the maintenance phase of infection.

### Sp100 represses late, but not early or intermediate, viral mRNA transcription in differentiated cells

Using CIN612-9E cells, we can also study the transition from the maintenance stage of the viral life cycle to the productive stage. Spink et al. demonstrated that differentiation activated the late viral promoter in CIN612-9E cells [12], and so we assessed the effect of siRNA depletion of PML and Sp100 on early, intermediate and late HPV mRNAs in the context of differentiation.

**Fig 6:**
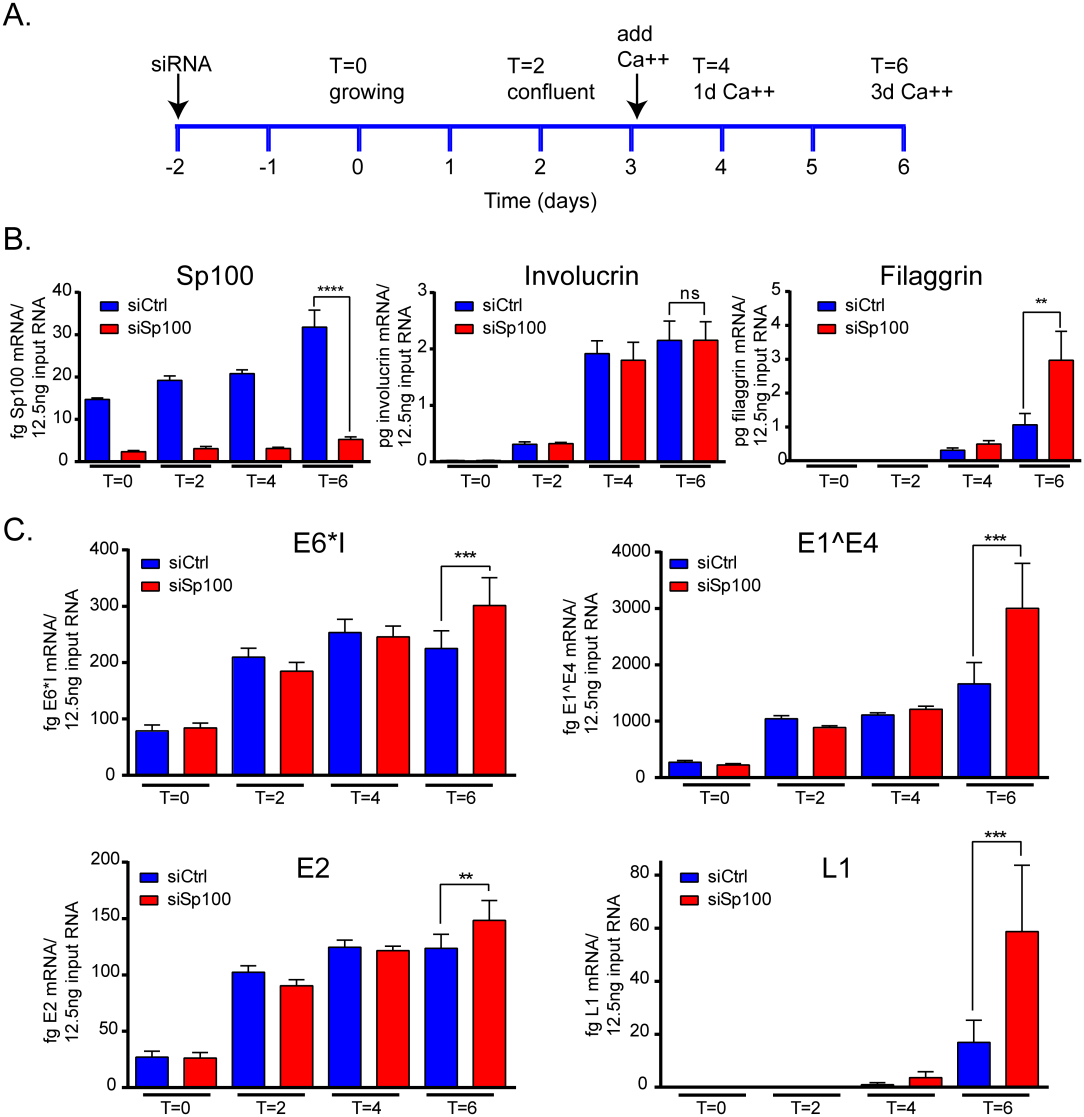
Sp100 represses late HPV mRNA transcription. **A.** Timeline of experiment. CIN612-9E cells were transfected with 20 nM siRNA to Sp100 and grown as shown. RNA was isolated at T = 0, 2, 4 and 6 days. **B.** Cellular transcripts for Sp100, involucrin and filaggrin were measured at the time points indicated. **C.** Viral transcripts were measured using qPCR with primers that overlapped the splice sites of E6*I, E2 877^2646, E1^E4 and L1. All results were obtained from three independent experiments, each of which contained two technical replicates. Error bars represent +/- SEM. *, P<0.05; **, P<0.005; ***, P<0.0005; ****, P<0.00005; ns, not statistically significant.

Proliferating CIN612-9E cells were transfected with siRNA to Sp100, grown to confluence and cultured in medium containing 1.5 mM CaCl_2_ for three days (see timeline in Fig 6A). siRNA depletion efficiency was monitored by qPCR for Sp100 using primers that detect all spliced transcripts for this gene (Fig 6B). At the end of the experiment (T = 6), Sp100 mRNA was still reduced 75–85% compared to siCtrl (Fig 6B). Differentiation was also monitored by the measurement of involucrin and filaggrin mRNA (Fig 6B). We observed a substantial inhibition of differentiation in siPML treated cells [15] and so these samples were excluded from further transcriptional analysis. Sp100 depletion did not affect involucrin levels, though filaggrin was observed to increase at later stages of differentiation (Fig 6B). Both filaggrin and involucrin are late keratinocyte differentiation markers [16], and so it is unlikely that Sp100 downregulation is globally promoting differentiation. Furthermore, it has been shown that activation of papillomavirus late gene transcription and genome amplification upon differentiation is coincident with involucrin expression [17]. However, we cannot completely rule out that Sp100 effects on differentiation are indirectly affecting viral late functions. Analysis of four different HPV31 RNA species showed small increases in early (E6*I; 1.2–1.5 fold), and intermediate (E2; 1.0–1.3 fold and E1^E4; 1.4–2.0 fold) in differentiated cells. However, consistently the late mRNA, L1 3590^5552, was elevated 3–12.4 fold in cells depleted for Sp100 compared to control treated cells (Fig 6C). We conclude that Sp100 most likely primarily represses late transcription.

### Sp100 represses viral genome amplification in differentiated cells

**Fig 7:**
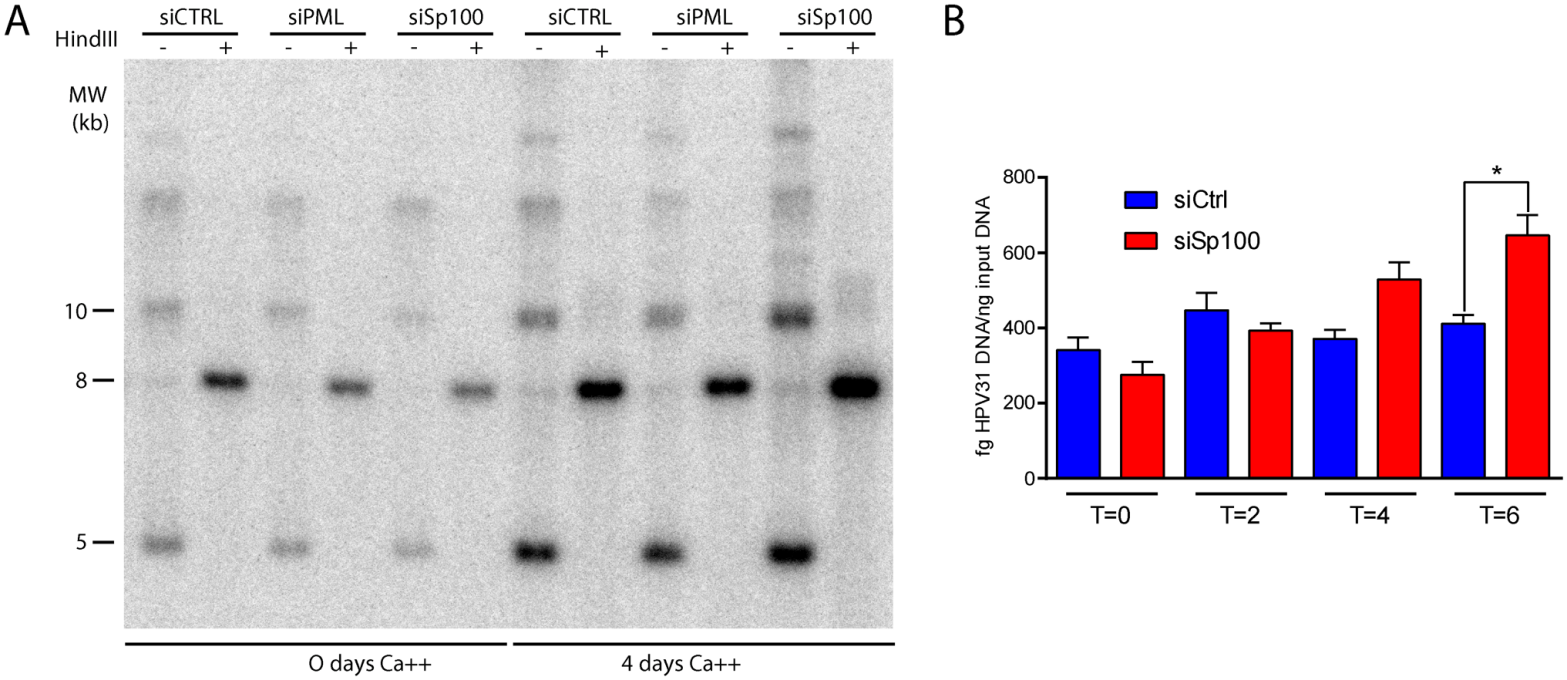
Sp100 represses differentiation dependent genome amplification. **A.** Southern blot analysis of 9E cell DNA extracted from cells treated with either control, PML or Sp100 siRNA for two days before addition of 1.5 mM CaCl_2_ to induce differentiation. DNA was collected at 0 or 96 hours post addition of CaCl2. Prior to electrophoresis, DNA was digested with a restriction enzyme that left supercoiled viral DNA intact (-), or with HindIII to linearize the viral genome (+). DNA was separated by agarose gel electrophoresis and probed with ^32^P-labeled HPV 31 genome. The blot shown is representative of four total Southern blots. **B.** DNA qPCR was performed on CIN612-9E cells treated according to the scheme shown in Fig 6A. Results shown were averaged from three independent experiments, each of which contained two technical replicates. Error bars represent +/- SEM. *, P<0.05.

To analyze the effect of Sp100 on HPV31 genome amplification, CIN612-9E cells were analyzed by qPCR and Southern blotting (Fig 7A and 7B). After Sp100 downregulation, the viral DNA copy number in differentiated cells was consistently increased. The magnitude of the increase was not high, probably because of the small percentage of cells that switch to the productive phase of viral replication after calcium treatment, but it was highly consistent. Taken together, these data suggest that Sp100 associates with viral replication factories to limit the viral DNA synthesized during genome amplification, and to repress transcription of late mRNAs transcribed from the newly amplified DNA.

### Characterization of Sp100 isoforms in primary human keratinocytes

Sp100 encodes several splice variants, commonly referred to as Sp100A, Sp100B, Sp100C and Sp100HMG [18] and S1 Fig. Each of these isoforms share a common N-terminal domain that promotes dimerization [19] and another that mediates interaction with heterochromatin via association with the Heterochromatin Protein-1 (HP-1) family of proteins [20]. Additionally, the Sp100B, C and HMG isoforms share a DNA binding motif known as the SAND (Sp100, AIRE-1, NucP41/45 and DEAF-1) domain that directly binds DNA [21]. Sp100C and—HMG also have additional DNA binding (HMG box) and chromatin interacting motifs (PHD and bromodomains) that further implicate Sp100 in regulation of cellular gene expression [18]. To determine which Sp100 isoforms were responsible for repression of HPV transcription and replication, we first measured the isoforms present in primary HFKs. Using primers specific to either Sp100A, B, C or HMG, we found that all four Sp100 isoform transcripts were expressed in primary human keratinocytes (S1 Fig, panel C). Sp100A mRNA was the most abundant isoform, followed by Sp100B mRNA. Sp100C and Sp100HMG mRNA were both detectable, but at levels four to fifteen times lower than Sp100A for Sp100C and Sp100HMG, respectively.

At the protein level, the Sp100 isoforms are predicted to migrate at approximately 52 kDa (Sp100A), 75 kDa (Sp100B), or 97 kDa (Sp100C and HMG). However, most studies detect only two predominant molecular weight species that migrate between 65–80 kDa and ~100 kDa when analyzed by SDS-PAGE and these species are frequently identified as either un-modified or SUMO-modified Sp100A [22]. To further characterize the Sp100 species present in HFKs, we transfected keratinocytes with expression vectors encoding EE-tagged versions of either Sp100A, B, C, HMG or an empty vector. The EE-tag adds only ten amino acids to Sp100 and thus can help delineate endogenous Sp100 isoforms. Immunoblot analysis with a pan-Sp100 antibody showed the same two predominant Sp100 species previously described (~65 kDa and ~80 kDa), as well as a low abundance of several higher molecular weight species (S1 Fig, panel D). Comparison of lysate from cells transfected with the Sp100A expression vector to the empty vector lysate revealed two major Sp100 protein species that migrated slightly above the two major endogenous Sp100 bands (S1 Fig, panel D). These are most likely unSUMOylated and SUMOylated forms of Sp100A (S1 Fig, panel D), while the species migrating slightly above 100 kDa is most likely Sp100A that has acquired two SUMO modifications [23]. Lysates from cells transfected with an Sp100B expression vector showed a major band migrating at ~100 kDa, and a minor species at ~115 kDa (S1 Fig, panel D). From the migration pattern, it seems that almost all endogenous Sp100B is SUMOylated. Lysates from cells transfected with either Sp100C or Sp100HMG expression vectors contained protein species that corresponded with the slowest migrating forms of endogenous Sp100 (S1 Fig, panel D). Because Sp100C and Sp100HMG have nearly identical molecular weights, we were unable to distinguish between them. In summary, all four major splice variants of Sp100 are present in primary human keratinocytes.

### Interferon upregulates all Sp100 splice variants in primary HFKs

Type I-IFN upregulates Sp100A and other, higher molecular weight Sp100 species in several human cell types [24–26]. Treatment of HFKs with IFN-α upregulated all Sp100 isoforms with peak induction of all isoform mRNAs six hours post-exposure (S2 Fig, panels A-F). The mRNA levels for Sp100A, -B, and -HMG increased approximately fivefold, while Sp100C mRNA levels were induced almost 10-fold over uninduced cells. We also examined the upregulation of Sp100 at the protein level following treatment with IFN. Upregulation of Sp100A and Sp100C/HMG (both un-modified and SUMO-modified) protein level was apparent 24 hours post-treatment and was sustained for at least 72 hours (S2 Fig, panel G). On examination of Sp100 in situ, we observed a substantial increase in both the size and number of PML and Sp100 nuclear foci at 48 hours post-IFN treatment (S2 Fig, panel H). These data demonstrate that the major isoforms of Sp100 are IFN-responsive in primary skin cells.

### The SAND domain containing isoforms of Sp100 repress HPV18 transcription and replication

**Fig 8:**
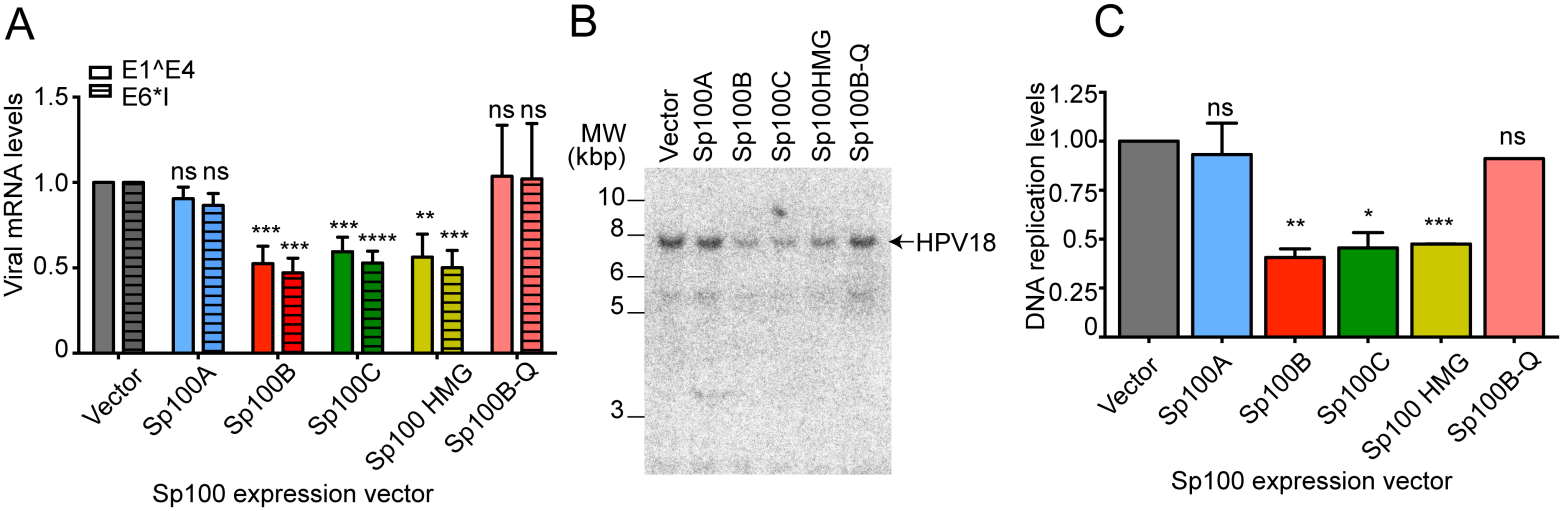
SAND domain containing isoforms of Sp100 repress viral transcription and replication. **A.** qPCR was performed on cDNA prepared from HFKs coelectroporated with 2μg of recircularized HPV18 genome and 1μg of an empty expression vector or a vector expressing either Sp100A, B, C, HMG or the Sp100B-DBD mt. Samples were harvested 72 hours post DNA transfection. The spliced viral messages E1^E4 (solid bars) or E6*I (hashed bars) were detected using specific primers for the spliced messages. Data displayed are averages of three independent experiments using four different HFKs donor strains (n = 4 replicates). All mRNA levels were normalized to TBP. **B.** Southern blot analysis of whole cell DNA extracted from the same coelectroporated cells graphed in A. DNA was separated by agarose gel electrophoresis and probed with ^32^P-labeled HPV18 genome 72 hours after DNA transfection. Prior to electrophoresis, DNA was digested with the restriction endonucleases DpnI to remove input, un-replicated DNA and EcoRI to linearize the viral genome. The arrow indicates the linear form of the viral DNA. Blot was performed using DNA from one of the two replicates shown in panel A. **C**. Phosphorimage quantitation of Southern blot assays from two independent experiments, except Sp100B-Q sample was tested only once. Error bars represent SEM. The paired student t-test was used to determine statistical significance between the EYFP-transfected control and each Sp100 isoform for panel A and C. n.s., not statistically significant; *, p<0.05; **, p<0.01; ***, p<0.001; ****, p<0.0001.

Sp100A has been shown to activate a CMV-promoter controlled gene array by promoting chromatin decondensation, and adenovirus retains Sp100A in viral replication centers while excluding the SAND domain containing isoforms to evade Sp100-mediated repression [27]. Habiger and colleagues have also shown that the three longer isoforms of Sp100 can repress luciferase expression from HPV18 and HPV31 URR (upstream regulatory region) driven reporter plasmids [8]. To determine whether individual Sp100 isoforms regulate transcription and replication from the HPV genome, HFKs were coelectroporated with HPV18 recircularized genomes and each of the Sp100 expression vectors. In cells coelectroporated with an Sp100A expression vector, only a small (and non-significant) reduction in HPV18 E1^E4 or E6*I transcription was observed (Fig 8). However, the SAND domain containing isoforms (B, C and HMG) reduced viral transcription by approximately 50–60%. A mutation in the DNA binding region of the SAND domain of Sp100B reduced transcriptional activity similar to empty vector (Fig 8A); however, as shown in S1 Fig, panel D, the steady state levels of the mutated Sp100B was less than that of wild type, making it difficult to conclude that the SAND domain is crucial for the observed repression. We also analyzed viral replication in the same transfected samples. No measurable change in HPV18 replication was apparent in cells cotransfected with Sp100A (Fig 8B), but the SAND domain containing isoforms of Sp100 repressed viral replication. Furthermore, in cells transfected with the expression vector encoding Sp100B containing the SAND domain DNA binding mutation, replication was restored to wild-type levels (Fig 8B). However, as described above, we could not absolutely conclude that repression required the SAND domain function because of reduced stability of the protein. In conclusion, the three longer isoforms of Sp100 repress HPV transcription and replication.

### Binding of Sp100 to viral chromatin increases in differentiated cells

**Fig 9:**
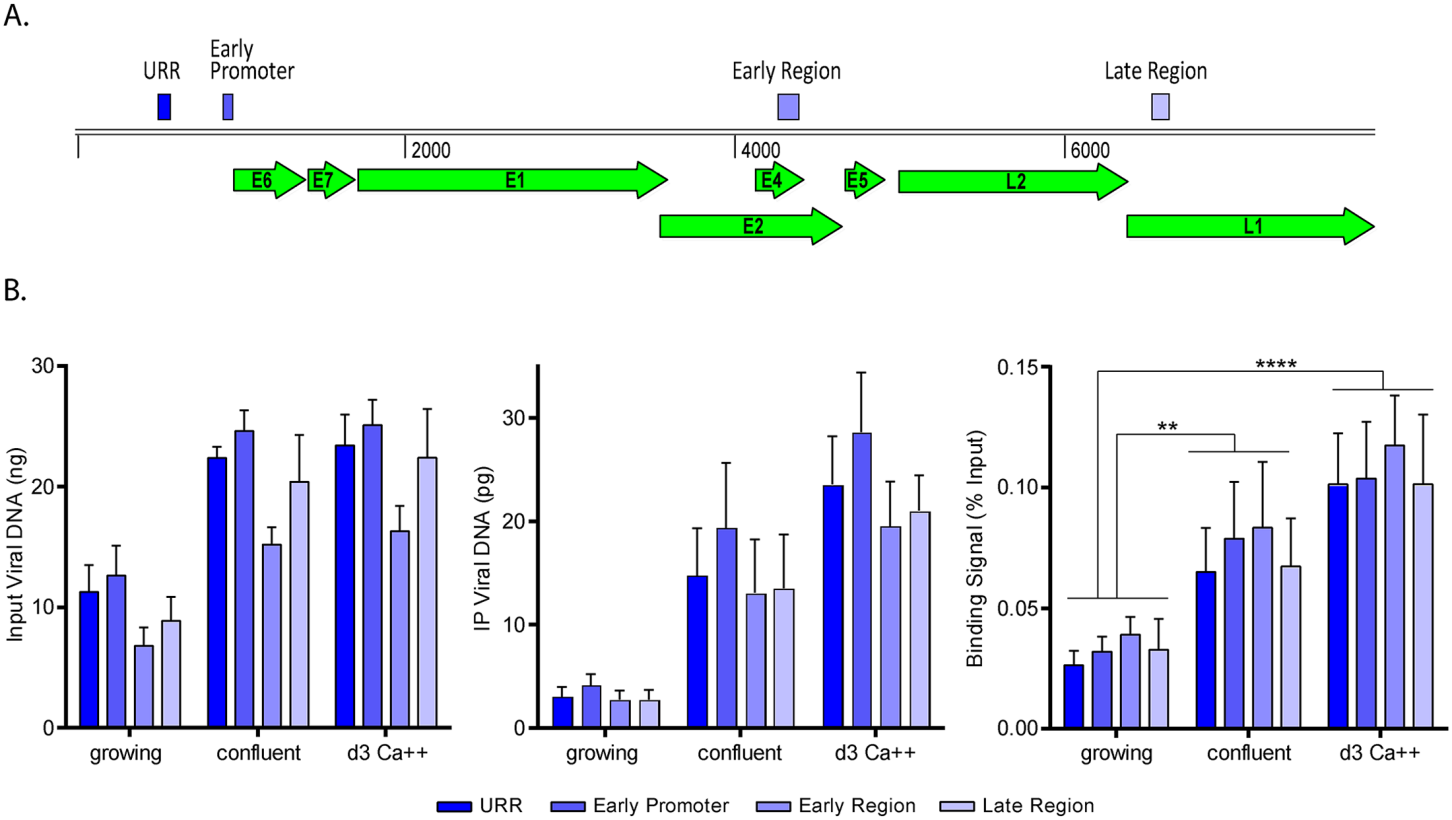
Sp100 binding to HPV chromatin correlates with differentiation-dependent viral functions. Chromatin immunoprecipitation (ChIP) was performed with samples from growing and differentiated CIN612-9E cells using rabbit IgG and Sp100 antibody. Viral DNA was quantified with qPCR using primers targeting major regions of the HPV31 genome. **A.** Linear representation of HPV31 genome displaying primer positions (orange boxes) for the upstream regulatory region, early promoter, early region, and late region. **B.** ChIP binding signals were expressed as a percentage of Sp100 coimmunoprecipitated viral DNA (minus that precipitated with a background IgG antibody) DNA relative to total viral DNA in ChIP input (% Input). Binding signals were averaged from three independent experiments, and the amounts shown are from the equivalent of 2 μg input chromatin. Error bars represent +/- SEM. Statistical significance was calculated using a paired Student t-tests. SD. **, P<0.005; ****, P<0.00005. S3 Fig shows Sp100 protein levels, and S4 Fig shows background IgG binding levels, and histone H3 levels.

Since the repressive forms of Sp100 contain both DNA binding and chromatin binding domains, we sought to determine whether Sp100 binds to viral chromatin to repress transcription and replication in differentiated 9E cells. Primers were designed to amplify different regions across the HPV31 genome, and those that passed qPCR quality control are shown in Fig 9A. To ensure that immunoprecipitation by the Sp100 antibody was specific, we treated confluent 9E cells with interferon to increase Sp100 expression (S3 and S4 Figs). This showed that the Sp100 isoforms are upregulated by interferon in confluent CIN612-9E cells (S3 Fig, panel A), in a pattern very similar to that of primary HFKs (S2 Fig). Chromatin was prepared from growing, confluent, and calcium treated 9E cells, as well as confluent 9E cells that had been treated for 24 hours with 25 ng/ml IFNα. Interferon increased the amount of Sp100 binding to HPV31 DNA by about threefold, indicating that the ChIP assay was specific. Additional controls using negative control antibodies (IgG), and positive control histone H3 antibodies are shown in S4 Fig. This showed that the Sp100 antibody coimmunoprecipitates up to 70-fold more viral DNA than the IgG negative control. To further prove the specificity of the Sp100 antibody, Sp100 protein was downregulated by siRNA before ChIP analysis. This showed that there was a very substantial decrease in the amount of viral DNA immunoprecipitated after siSp100 treatment (S4 Fig panels B and C). Therefore, the Sp100 ChIP assay is robust, and specific.

Collectively, the ChIP analyses showed that Sp100 bound to all regions of the viral genome (similar to histone H3); no specific region of the genome was targeted by Sp100 and so its repressive effect is viral sequence independent. We compared Sp100 binding to the HPV31 genome in growing, confluent, or differentiated 9E cells (treated for three days in high CaCl_2_ medium after reaching confluence). Viral DNA copy number increased when cells reached confluency but did not increase further after culture in CaCl_2_ medium (Fig 9). We saw a pronounced increase in the amount of viral DNA coimmunoprecipitated with Sp100 as cells progressed from growing to confluence, and again after CaCl_2_ treatment. When the percentage of genome bound by Sp100 was calculated (% input), it was observed that Sp100 binding increased both when viral DNA was amplified at cellular confluence and when differentiation was further increased after CaCl_2_ treatment. Overall, there was a 2.6 to 4.9 fold increase in Sp100 binding per viral genome upon differentiation (Fig 9). Therefore, Sp100 binding to the HPV genome increases as cells transition into the late, productive stage of infection.

To obtain a more exact quantitation of the increase in Sp100-viral DNA association upon differentiation, we performed an absolute quantification of Sp100 protein and HPV31 DNA in chromatin immunoprecipitates from growing and differentiated 9E cells. The Sp100 protein content in input and immunoprecipitated chromatin samples was calculated by reference to a standard curve of in vitro translated Sp100 protein, and the absolute quantity of HPV31 genomes in each sample was measured through ChIP-qPCR analysis of input DNA and immunoprecipitated viral DNA against an HPV31 plasmid DNA standard curve. As shown in S5 Fig, there was ~ eightfold increase in the number of HPV31 genomes associated with Sp100 in differentiated cells, taking into account decreased Sp100 protein and increased viral DNA levels observed in input chromatin fractions after differentiation.

Sp100 binding to viral DNA also increased substantially with interferon treatment (S4 Fig). The viral DNA copy number showed only a small decrease after IFN treatment, as measured from input chromatin samples (S4 Fig) and so the observed increase in binding reflected increased Sp100 binding per viral genome. The increase in binding in response to IFN treatment supports the hypothesis that Sp100 binding to HPV31 DNA is an anti-viral response.

Interestingly, the amount of histone H3 bound to viral DNA increased with differentiation. This could be due to increased chromatin accessibility in the rapidly amplifying viral DNA in replication foci, or changes in topological restraints, as the genomes switch from bidirectional theta replication to recombination-directed replication [28]. We have previously shown that there are different patterns and enrichments of modified histones in the replication foci in 9E cells [13] and follow-up studies will analyze the role of chromatin dynamics in late viral replication.

### Sp100 is expressed in the stratum spinosum of the cervical epithelium and is upregulated in HPV infected lesions

For Sp100 to repress late HPV transcription, it must be expressed in cells that amplify viral DNA and transcribe late mRNAs. Nakahara and Lambert examined PML bodies in organotypic raft tissue and showed that the number of bodies were increased in HPV-infected cells and tissue [29]. They showed that the PML bodies were present in basal and suprabasal cells, but were no longer present in the most differentiated cells. To examine if the same is true for Sp100, we stained sections of tissue derived from an HPV16-infected cervical lesion with antibodies to Sp100 and E4, and combined this with FISH for viral DNA. We noted that, as described for PML by Nakahani and Lambert, Sp100 expression was increased in HPV-infected tissue compared to normal tissue. Foci of Sp100 were observed in basal cells and these increased throughout the stratum spinosum, but disappeared in the most differentiated cells. S6 Fig shows single cell quantitation of Sp100 expression in normal and HPV16-infected cervical tissue that confirms these findings.

**Fig 10:**
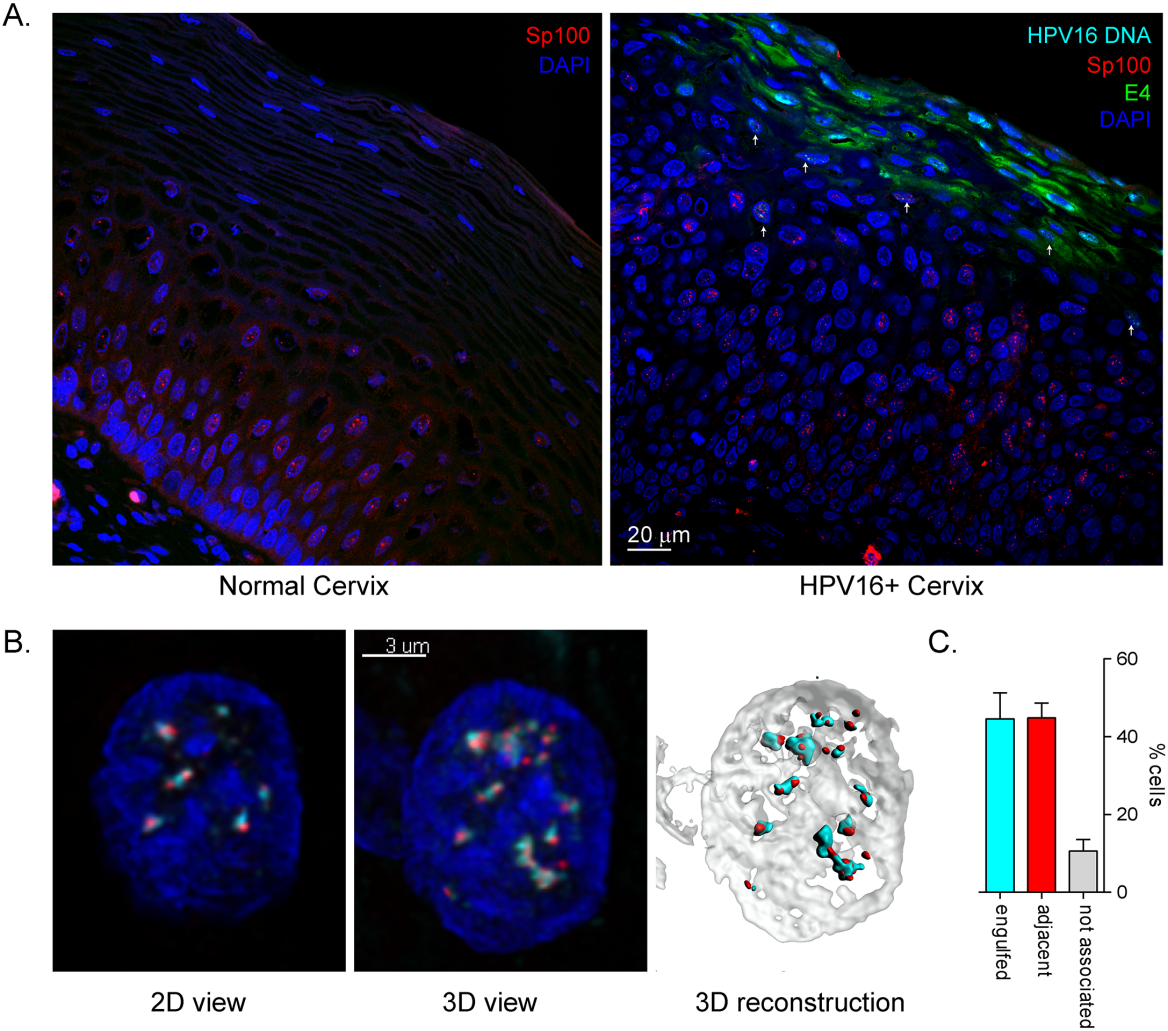
Viral DNA amplification initiates in cells containing Sp100 foci. Normal cervix and CIN-I HPV16-infected cervical tissue sections were subjected to antigen-retrieval followed by combined FISH-IF (Red: Sp100, Cyan: HPV16 DNA, Blue: DAPI, Green, E4) for confocal microscopy analysis. A. Images showing full thickness sections of stained cervical epithelia. Arrows indicate examples of cells containing both amplifying HPV genomes and Sp100 foci. B. Left, 2D view of a single focal plane of a keratinocyte at the onset of viral DNA amplification with pronounced Sp100 colocalization. Center, 3D view of Z-stack from the same image. Right, 3D reconstruction from the Z stack with rendered surfaces for nucleus (Gray, DAPI), Sp100 (Red), and HPV16 DNA (Cyan) showing Sp100 foci adjacent and surrounded by HPV16 DNA. C. Quantitation of experiment shown in B. 3D images of cells containing both Sp100 foci and HPV DNA were visually assessed and the association of Sp100 foci and HPV DNA signal categorized as not associated, adjacent, or engulfed (Sp100 foci surrounded by HPV DNA signal). Results are from two independent experiments with a total of N = 76 cells counted.

Viral DNA amplification occurs in the upper layers of HPV16-infected tissue and we wanted to determine whether this occurs in cells that still express Sp100. As shown in Fig 10A, viral DNA amplification was initiated in cells that still contained Sp100 foci. In the layer of cells above this, full-blown DNA amplification could be observed and Sp100 was no longer present. A careful analysis and 3D reconstruction of cells initiating viral DNA amplification in the presence of Sp100 showed that in the majority of cells (~90%) Sp100 foci were localized adjacent to, or engulfed by, HPV replication foci (Fig 10B and 10C). Therefore, Sp100 foci and viral DNA replication foci interact at late times of infection, concomitant with viral DNA amplification and late gene transcription.

## Discussion

In this study, we found that HPV replication factories generated by expression of the E1 and E2 replication proteins, and those formed in differentiated cells containing the HPV genome, are associated with PML and Sp100 containing ND10 bodies. The ND10 bodies were mostly observed adjacent to, or in a satellite pattern around, the replication factories; however, PML and Sp100 proteins could also be observed inside large foci in close association with amplifying viral DNA. In cells containing E1-E2 generated replication foci, the normal, nuclear body staining pattern of Sp100 and PML was disrupted, and in many cases all nuclear Sp100 had accumulated into a single replication factory. Sp100-containing nuclear foci were also detected adjacent to viral replication foci in the upper layers of HPV-infected cervical lesions, and often single or multiple Sp100 foci were engulfed in a cloud of viral DNA. The role of Sp100 was analyzed in detail; we determined that Sp100 was associated with viral chromatin, and this association increased concomitant with the formation of replication foci in differentiated cells, resulting in repression of viral transcription and replication. Transient expression of the individual isoforms of Sp100 showed that the three longer isoforms of Sp100 (Sp100B, C and HMG) could limit viral replication and transcription.

Sp100 proteins limit the transcription and replication of many viruses, often by repressing incoming genomes at the early stages of infection [7, 8, 26, 27, 30–35]. The replication factories that form in the late, productive phase of several viruses are also associated with ND10 bodies and late viral functions can also be specifically repressed by Sp100 [30, 36, 37]. We have shown previously that Sp100 represses viral transcription, replication and establishment at early times after infection [7], and here we extend these findings to show that the differentiation-dependent, productive stage of HPV infection is also limited by Sp100.

Analysis of ND10 protein modulation of viral processes can be challenging because different components can have opposing effects [37, 38], and many viruses encode antagonists that negate anti-viral effects [38]. Moreover, both PML and Sp100 encode several different isoforms that differentially modulate viral transcription and replication. An additional challenge in studying the late phase of the HPV life cycle is that it is dependent on differentiation of the host keratinocyte. We successfully downregulated Sp100 in differentiated CIN612-9E cells, but siRNA-mediated repression of PML expression inhibited keratinocyte differentiation and so we were unable to further analyze the role of PML in differentiated cells. We also observed some effect of siRNA-mediated repression of Sp100 on filaggrin, but not involucrin, keratinocyte differentiation markers, and so there remains the possibility that some of the effects of siSp100 observed on late viral functions are indirect. Calcium induction of differentiation in HPV31 containing CIN612-9E cells induces viral DNA amplification and late gene transcription, though it is not possible to achieve the magnitude of viral DNA and RNA observed in vivo. Nevertheless, enough viral DNA amplification and late gene expression are induced to allow the effect of Sp100 to be measured. Repeated experiments showed that Sp100 depletion had only minimal effect on viral early or intermediate mRNA transcripts, but consistently upregulated late L1 transcripts and increased viral DNA amplification (Figs 6 and 7). This identifies a role for Sp100 in the productive stages in the viral lifecycle. Similarly, Sp100 has an anti-viral effect at both early and late stages of infection by hCMV [30], and HSV [39]. Reactivation of EBV lytic infection also results in the association of EBV genomes and viral gene products with ND10 body components [36]. In the current study, we also observed Sp100 binding to viral chromatin and showed that this binding increased with differentiation. Thus, Sp100 likely functions as a repressor of late viral transcription and replication, providing infected cells with a late-stage anti-viral activity.

We have previously shown that depletion of Sp100 during maintenance replication has only minimal effects on viral DNA copy number or viral transcription [7], and we confirm that finding here for HPV31 containing-cells. Thus, Sp100 repression seems to be mostly confined to early and late stages of viral genome amplification. We speculate that this could be due to the unlicensed, unscheduled viral DNA replication that takes place at these stages. Viral DNA amplification is closely associated with the cellular DNA damage and repair response (DDR)[40], and components of the DDR interact with or localize to ND10 [41–43]. The host DDR is important for amplification of viral DNA in differentiated cells [44, 45] and the recruitment of DNA repair factors to replication factories within these cells is thought to facilitate viral DNA replication in cells that have exited the cell cycle and are no longer replicating the host DNA [28, 46]. This suggests a complex relationship between late-stage HPV genome amplification, the DDR, and the intrinsic immune system. We also speculate that only viral transcription from templates that have undergone amplificational replication are subject to significant Sp100 repression. This would explain why early viral mRNA transcripts (such as E6*I) are repressed at early stages of infection [7], while primarily late transcripts (L1) are repressed in differentiated cells (this study).

We further examined the HPV-Sp100 interaction by characterizing the Sp100 isoforms expressed in primary keratinocytes to elucidate which isoforms could contribute to Sp100-mediated repression of HPV infection. We observed all four major Sp100 isoforms expressed in primary HFKs with Sp100A being the most abundant isoform at both the RNA and protein level (S1 Fig). Furthermore, IFN-α enhanced expression of all four isoforms (S2 Fig) in a pattern very similar to that described for BJ fibroblasts [26]. In both cell types, Sp100C was expressed at relatively low levels compared to the other isoforms but was most strongly induced at the transcriptional level by IFN-α. Habiger et al., also recently showed that most Sp100 isoforms are expressed at reduced levels in CIN 612-9E cells, compared to primary HFKs [8], consistent with the global downregulation of interferon-induced genes in HPV-infected cells [47]. Notably, all Sp100 isoforms observed by immunoblot analysis could be induced by exogenous IFN in HPV31-containing differentiated 9E cells (S3 Fig). We also observed an increase in the SUMOylation of Sp100A upon differentiation (S3 Fig). However, we were unable to analyze the role of the Sp100 isoforms in differentiating CIN612-9E cells (siRNAs against the individual isoforms showed substantial crosstalk). Nevertheless, by cotransfecting expression vectors for each isoform along with an HPV18 viral genome into keratinocytes, we found that expression of the SAND domain containing isoforms Sp100B, C and HMG significantly reduced viral gene expression and DNA replication (Fig 8). Mutation of the DNA binding domain of Sp100B (Sp100B-Q) abrogated this repression, but this result has to be interpreted with caution as the mutated protein had decreased steady state levels. A similar result was obtained by Habiger and colleagues who found that the three longer forms of Sp100 could repress transcription from an HPV URR driven reporter plasmid [8]. Correspondingly, the SAND domain containing isoforms of Sp100 also function as repressors of HSV immediate-early transcription [25, 26] and adenovirus infection [27]. In the latter case, Sp100A is recruited to adenoviral replication centers, while the repressive isoforms (B, C and HMG) are redistributed away from these sites [27]. Sp100A can decondense viral chromatin and activate viral transcription in the context of CMV infection [48], while Sp100B binds DNA and represses transcriptional activity of DNA containing unmethylated CpG motifs [49, 50]. Therefore, the different Sp100 isoforms differentially regulate transcription and replication of several viruses.

All four isoforms of Sp100 contain a motif that promotes interaction with ND10 bodies, but the three longer isoforms contain additional domains that bind DNA and chromatin [49, 51]. This would be consistent with our finding that Sp100 colocalizes with, and binds to, viral chromatin. We did not observe specific association with any region of the viral genome and this could indicate that Sp100 is marking the genome for silencing rather than functioning at specific transcriptional elements or the viral replication origin.

It has previously been shown that PML containing ND10 bodies are increased in the poorly differentiated layers of HPV-infected organotypic rafts compared to uninfected rafts, but are absent in the most superficial, differentiated layers [29]. Sp100 containing ND10 bodies have also been detected in HPV33-infected cervical tissues and similarly are absent in the upper layers of tissue [6]. This raised the question as to whether Sp100 was actually present in differentiating cells that are amplifying viral DNA. However, we show that there is a clear transition zone where Sp100 is still expressed and viral DNA amplification begins. Furthermore, nuclear foci of Sp100 (likely ND10 bodies) are very frequently located adjacent to, or engulfed by, the replicating DNA. In summary, Sp100 associates with replication foci at late times in infection and appears to repress this stage of infection by binding to viral chromatin.

We have concluded that Sp100 functions as a repressor of late viral transcription and replication to provide infected cells with a late-stage anti-viral activity. However, there is also the strong possibility that HPV usurps the anti-viral functions of Sp100 to modulate the viral lifecycle. For example, Sp100-mediated repression could promote the shift from early infection to the maintenance phase of infection. Repression and condensation of viral chromatin at the late stage of infection could also prepare viral genomes for packaging in virion particles. Most likely, Sp100 attempts to restrict late viral infection, but HPV antagonizes this process, and probably hijacks some of its functions. This could explain the relatively modest effects of Sp100 we observe on late viral replication and transcription.

In summary, we have shown that Sp100 represses viral replication and transcription at early and late stages of infection. Therefore, the host intrinsic immune system functions to restrict viral activity in newly infected cells, but is also activated when persistently infected cells switch into the productive stage of the viral life cycle upon differentiation. We predict that this is linked to the unscheduled viral DNA amplification that takes place at these times.

## Materials and methods

### Plasmids: HPV genomes, replicons and expression vectors

Wildtype genomes for HPV16, HPV18 and HPV 31 cloned in prokaryotic vectors have been described previously [52–54]. The mammalian expression vectors for N-terminally glu-glu (EE) epitope tagged HPV16 E1 (pMEP9-HPV16EE-E1), and N-terminally FLAG epitope tagged HPV16 E2 (pMEP4-HPV16FLAG-E2) have been described previously [55, 56]. pKS(-) and pKS-HPV16 ori plasmids have been described previously [57].

EYFP-tagged versions of Sp100A, Sp100B, Sp100C, Sp100HMG, and Sp100B-W655Q were obtained from Susan Janicki (Wistar Institute; Philadelphia, PA) and have been described previously [48]. N-terminal, EE epitope-tagged versions of each Sp100 isoforms were generated by restriction digestion of pEYFP-Sp100A, pEYFP-Sp100B, pEYFP-Sp100C, pEYFP-Sp100HMG, pEYFP-Sp100B-W655Q with NheI and XhoI to remove the N-terminal EYFP tag and it was replaced with a synthesized DNA sequence containing a T7/SP6 promoter, β-actin mRNA leader sequence, and an EE epitope tag (GeneART; Hamburg, Germany). The resulting plasmids were named pCMV-TnT-Sp100A, -Sp100B, -Sp100C, -Sp100HMG and -Sp100B-W655Q, respectively.

### Viral genome recircularization

To obtain recircularized HPV genomes, 10 μg HPV DNA, cloned in a prokaryotic vector, was digested with a restriction enzyme to release the viral DNA. Following digestion, the restriction enzyme was either heat inactivated or purified away from the digested DNA using HiPure PCR Purification Columns (Roche AG; Mannheim, Germany). The released viral DNA was resuspended at 10 μg/ml in 1X ligation buffer (66 mM Tris-HCl, pH 7.5, 5 mM MgCl_2_, 5 mM DTT, 1 mM ATP) with 40 units of T4 DNA ligase (New England BioLabs; Ipswich, MA) in a total volume of 900 μl and incubated at 16°C for 12–18 hours. Following ligation, NaCl was added to the solution to a final concentration of 0.5 M and the DNA was precipitated with 0.6 volumes isopropanol. Precipitated DNA was washed two times with 70% EtOH and resuspended in 1X TE buffer. The efficiency of viral genome religation was determined by separation of 200 ng pre- and post-ligation using DNA agarose gel electrophoresis. The final dsDNA concentration was determined by spectrophotometric analysis using the Nanodrop-1000 (Life Technologies; Forest City, CA).

### Cell culture

The conditionally immortalized human foreskin keratinocytes (HFK1a) was developed in our laboratory and has been described previously [56] and was cultured in F-medium supplemented with the Rho-kinase inhibitor, Y-27632 (Tocris Bioscience; Bristol, United Kingdom) at a final concentration of 10 μM. CIN612-9E cells, a cell line derived from a CIN1, HPV31-positive patient biopsy, that harbors extrachromosomal HPV31 genomes [9], were grown in F-medium without antibiotics and was provided by Lou Laimins (Northwestern University). Primary human keratinocytes were isolated from neonatal foreskins, as described previously [58]. Keratinocytes from each donor were independently designated HFK strain #.

Cells were cultured in Rheinwald-Green F-medium (3:1 Ham’s F12/DMEM-high glucose, 5% fetal bovine serum, 0.4 μg/ml hydrocortisone, 8.4 ng/ml cholera toxin, 10 ng/ml epidermal growth factor (EGF), 24 μg/ml adenine, and 6 μg/ml insulin) on a layer of lethally irradiated NIH J2 3T3 murine fibroblasts [59]. Antibiotics were not used unless otherwise noted. NIH J2 3T3 mouse fibroblast cultures (obtained from Craig Meyers, Penn State University) were maintained at low passage and cultured in DMEM/10% newborn calf serum. Prior to coculture with human keratinocytes or CIN612-9E cells, feeders were exposed to 60 grays of γ-irradiation using a Model 30 J.L Shepard Cs-137 Mark I irradiator (San Fernando, CA).

For differentiation assays, CIN612-9E cells were transferred to low calcium basal medium (Lonza Corporation; Walkersville, MD) supplemented with SingleQuots for keratinocytes (Lonza Corporation; Walkersville, MD) containing bovine pituitary extract, hydrocortisone, and epidermal growth factor, grown until confluent, and then cultured for 96 hours in basal medium Lonza Corporation; Walkersville, MD), supplemented with 1.5 mM calcium chloride (Lonza Corporation; Walkersville, MD). To induce Sp100 expression in ChIP assays, cells were cultured for 24 hours with 25 ng/ml carrier-free, human interferon α A (PBL Assay Science; Piscataway, NJ) in low calcium basal medium described above.

### DNA transfections

HFK 1A cells cultured on glass coverslips in F-medium supplemented with 10 μM Y-27632 were transfected with HPV16 E1 and E2 expression vectors (400 ng each) and 50 ng of pKS (empty vector), pKS-16Ori, or recircularized HPV16 genome. DNA was introduced into cells using Fugene 6 (Promega Corporation; Madison, WI). Fugene 6 was mixed 3:1 (volume/mass) with target DNA in serum-free Ham’s F-12 nutrient mixture for approximately 30 minutes at room temperature. Following incubation, lipid:DNA complexes were added dropwise to cells and incubated until cell harvest. E1 and E2 expression was induced 24 hours after DNA transfection with 3 μM CdSO_4_ for four hours and cells were fixed with 4% PFA for confocal microscopy analysis by indirect immunofluorescence.

For cotransfection of HPV18 DNA with Sp100 isoform expression vectors, 2 μg HPV18 DNA and 1 μg Sp100 isoform DNA was incubated with 100 μl of room temperature Amaxa Nucleofection solution for 5 minutes as described in Fig legends. 10^6^ cells were gently mixed with the DNA/Nucleofection solution and transferred to an electroporation cuvette. Electroshock was delivered using the pre-programed setting, T-007. Cells were transferred to recovery medium (F-Medium with 10% fetal bovine serum and no EGF) and plated onto irradiated murine J2/3T3 fibroblasts.

### siRNA transfections

For RNA extractions, CIN612-9E cells were plated at a density of 1.3-8x10^4^ cells/cm^2^ onto plates containing 1.3–1.5 x10^4^ cells/cm^2^ of lethally irradiated J2/3T3 fibroblasts and cultured overnight. For ChIP experiments, CIN612-9E cells were cultured at a density of 4000 cells/cm^2^ on 15 cm plates containing 1.3–1.5 x10^4^ cells/cm^2^ of lethally irradiated J2/3T3 fibroblasts. Pools of four siRNA duplexes that target PML (L-006547-00-0010), Sp100 (L-015307-00-0005) or a non-targeting control siRNA (D-00181a0-10-20) were purchased from Dharmacon (GE Healthcare). siRNA transfections were performed as per manufacturer’s protocol. Briefly, siRNAs were complexed with Lipofectamine RNAiMAX transfection reagent (Thermo Fisher Scientific) in OptiMEM and added to cells with fresh F-medium at a final concentration of 20 nM. Cells were incubated with siRNA for indicated time periods before harvesting or further experimentation. All siRNA sequences are shown in S2 Table.

### RNA extraction and qPCR detection of viral transcripts

For RNA extraction from keratinocyte coculture experiments, feeder cells were removed using Versene (Life Technologies; Forest City, CA) prior to harvest. Total RNA was isolated using the RNeasy Mini-RNA extraction kit (Qiagen; Germantown, MD). RNA concentrations were determined using the NanoDrop 1000 spectrophotometer (Life Technologies; Forest City, CA). RNA integrity analysis was performed by capillary electrophoresis using RNA 6000 Nano kits (Agilent Technologies; Forest City, CA) on a 2100 Bioanalyzer system (Agilent Technologies; Forest City, CA). Reverse transcription reactions were performed with the Transcriptor First-Strand Synthesis kit (Roche AG; Mannheim, Germany) using 1 μg of total RNA, 60 μM random hexamers, and 2.5 μM oligo-dT primers and expression of the indicated genes was analyzed by qPCR using an ABI Prism 7900HT Sequence Detector or the QuantStudio 7 Flex Real Time PCR System (Applied Biosystems; Forest City, CA) using SYBR green PCR master mix (Roche AG; Mannheim, Germany). Each reaction mixture contained 1× SYBR green master mix, cDNA from 1 μg of RNA, and 0.3 μM each oligonucleotide primer in a total volume of 20 μl. In each run, a 10-fold dilution series (2.5x10^5^-2.5x10^-2^ fg) of the target mRNA standard was included to generate a curve of threshold cycle (Ct) versus log_10_ quantity (fg). DNA standards for the HPV31 mRNAs E1^E4 (nt 857–877^3294–3296), E6*I (nt 186–210^413–416) and L1 (nt 3562–3590^5552–5554) were commercially synthesized and cloned into the SfiI site of pMAT (GeneART; Hamburg, Germany). A DNA standard for the intermediate HPV31 E2 mRNA (nt 862–877^2646–2648) was generated by PCR amplification of HPV31 nucleotides 862–877^2646–2712 from cDNA derived from an HPV31-containing cell line and ligation of the PCR product into the cloning vector, pCR2.1, using a TopoTA cloning kit (Life Technologies; Forest City, CA). DNA standards containing amplicons for the cellular mRNAs Sp100A, Sp100B, Sp100C and Sp100 HMG were commercially synthesized and cloned into the SfiI site of pMAT (GeneART; Hamburg, Germany). DNA standards for the cellular mRNAs of involucrin, filaggrin and cyclophillin A were generated by PCR amplification of involucrin, filaggrin and cyclophilin A cDNAs derived from primary human keratinocytes using commercially available primers from Qiagen (Germantown; MD). pRSV-neo was purchased from ATCC (Manassas, VA) and has been described previously [60]. pDRIVE-GAPDH and pCMV-SPORT6-TBP were purchased from OpenBiosystems (Pittsburgh; PA). The β-actin-GFP plasmid containing the human β-actin promoter upstream of the actin-GFP sequence was purchased from AddGene (Cambridge; MA) and has been described previously [61].

Gene expression was measured and the data are reported as the quantity of specific cDNA present in cDNA prepared from 12.5–50 ng of RNA (labeled as quantity of RNA). The specificity of each primer pair was determined by dissociation curve analysis and the presence of single amplicons was confirmed by capillary electrophoresis using DNA1000 LabChips (Agilent Technologies; Forest City, CA) on a 2100 Bioanalyzer system (Agilent Technologies; Forest City, CA) or standard gel electrophoresis using 2.5% agarose-Tris acetate EDTA (TAE). Primer sequences are shown in S1 Table.

### DNA qPCR for viral genome copy number

For all keratinocyte coculture experiments, feeder cells were removed using Versene (Life Technologies; Forest City, CA) prior to harvest. Total cellular DNA was isolated from keratinocytes using the DNeasy Blood and Tissue Kit (Qiagen; Germantown, MD) at specific times of infection. 1 to 5 ng of DNA was analyzed by qPCR using 300 nM primers and SYBRgreen Master mix (Roche AG; Mannheim, Germany). Reactions conditions consisted of a 15 minute 95°C activation cycle, 40 cycles of 10 s 95°C denaturation and 30 s 60°C annealing and elongation. Copy number analysis was completed by comparing the unknown samples to standard curves of linearized HPV18 DNA. The DNA copy number of β-actin was used as an endogenous control for all DNA qPCR experiments. qPCR for HPV31 DNA copy number was performed following isolation of DNA from CIN612-9E cells. DNA was digested with HindIII and BamHI and amplified using HPV31-specific primers under the reaction conditions described above. All qPCR was performed using an ABI 7900HT PCR system or the QuantStudio 7 Flex Real Time PCR System (Applied Biosystems; Forest City, CA). Primers for all PCR reactions are listed in S1 Table.

### Southern blot analysis

For all keratinocyte coculture experiments, feeder cells were removed using Versene (Life Technologies; Forest City, CA) prior to harvest. Total DNA was harvested using the DNeasy Blood and Tissue Kit (Qiagen; Germantown, MD). At least 1 μg total DNA was digested with either a single-cut linearizing enzyme (HPV16, BamHI; HPV31, HindIII) for the HPV genome, or with a non-cutter (HPV16, HindIII; HPV31, BamHI) to linearize cellular DNA. After digestion, samples were separated on 0.8% agarose-TAE gels. DNA was visualized with 0.5 μg/ml ethidium bromide and was transferred onto nylon membranes using a Turbo Blotter downward transfer system (GE Healthcare; Pittsburg, PA). Membranes were UV cross-linked (120 mJ/cm^2^), dried and blocked with hybridization buffer (3X SSC, 2% SDS, 5X Denhardt’s solution, 0.2 mg/ml sonicated salmon sperm DNA) for 1 hour. After blocking, the membrane was incubated overnight with 25ng (^32^P)-dCTP labeled probe (specific for HPV16 or HPV31) in hybridization buffer (3X SSC, 2% SDS, 5X Denhardt’s solution, 0.2 mg/ml sonicated salmon sperm DNA). Hybridized DNA was visualized and quantitated by phosphor-imaging on a Typhoon Scanner (GE Healthcare; Pittsburgh, PA). To generate HPV-specific radiolabeled probes, linear viral DNA was cleaved from vector sequences and gel-purified using QiaQuick Gel Extraction kits (Qiagen; Germantown, MD). Radiolabeled probe was generated from 50 ng gel-purified linear HPV16 or HPV31 DNA with the Random Prime DNA Labeling Kit (Roche AG; Mannheim, Germany).

### Indirect immunofluorescence

Cells were cultured on #1.5 18 mm glass coverslips and fixed with 4% paraformaldehyde/ PBS. Fixed cells were permeabilized with 0.1% Triton X-100 in PBS and blocked in 5% (v/v) normal donkey serum (Jackson Immunoresearch; West Grove, PA). All primary antibody incubations were performed at 37°C for 1 hour. Antibodies used were: goat anti-PML A-20 (Santa Cruz Biotechnology, Dallas, TX; dilution 1:50); rabbit anti-PML (Sigma Corporation; St. Louis, MO; dilution 1:50); mouse anti-PML, PG-M3 Santa Cruz Biotechnology, Dallas, TX; dilution 1:50); rabbit anti-Sp100 (Sigma Corporation; St. Louis, MO; dilution 1:500); chicken anti-EE (Bethyl Laboratories; Montgomery, TX; dilution 1:100); mouse anti-FLAG M2 (Sigma Corporation; St. Louis, MO; dilution 1:500). Following primary antibody incubation, coverslips were washed three times in PBS. All secondary antibody incubations were performed at 37°C for 30 minutes. Alexa 488, Alexa 564, Alexa 594, Rhodamine Red-X or Alexa 647 conjugated to the desired species’ immunoglobulin was purchased from Jackson Immunoresearch (West Grove, PA) and were used at 1:100 dilution. Coverslips were mounted using 10 μl ProLong Gold with DAPI (Life Technologies; Forest City, CA). Cells were visualized using a Leica TCS-SP5 laser scanning confocal microscope (Leica Microsystems; Buffalo Grove, IL).

### Combined fluorescent in situ hybridization-Immunofluorescence (FISH-IF)

Cells were fixed and stained as described for IF. After antigen staining, cells were fixed again with methanol: acetic acid (3:1, v/v) at room temperature for 10 minutes followed by 2% PFA for 1 minute at room temperature to fix the antibodies in place. Cells were treated with RNace-iT cocktail (Agilent Technologies; Forest City, A). at a dilution of 1:1000 for 1 hr at 37°C and subsequently dehydrated in 70%, 90%, and 100% ethanol for 3 minutes each and air dried. HPV16 or 31 DNA FISH probe was prepared using the FISH-Tag DNA Multicolor Kit labeling kit (Life Technologies; Forest City, CA). DNA was conjugated to either Alexa 488 or Alexa 555, according the manufacturer’s protocol. Hybridization was performed overnight in 1X Hybridization Buffer (Empire Genomics; Buffalo, NY) with 20–75 ng of labeled probe DNA at 37°C. Slides were washed at room temperature with 1X phosphate-buffered detergent (PBD) (MP Biosciences, Inc; Cleveland, OH), followed by 1X wash buffer (0.5X SSC, 0.1% SDS) at 65°C, and a final wash with 1X PBD at room temperature. Coverslips were mounted using Prolong Gold with DAPI (Life Technologies; Forest City, A). and analyzed by confocal microscopy.

### Immunoblot analyses

For all keratinocyte coculture experiments, feeder cells were removed using Versene (Life Technologies; Forest City, A) prior to harvest. Whole cell lysates were extracted from primary human keratinocytes using SDS-extraction buffer (SDS-EB) (50 mM Tris, pH 6.8; 10% glycerol, 2% SDS) supplemented with cOmplete ULTRA protease inhibitor cocktail (Roche AG; Mannheim, Germany). 1X SDS-EB was added to each sample (200 μl/3.8 cm^2^ of culture dish area) and the lysate was collected by scraping. To shear genomic DNA, samples were sonicated using a cup horn sonicator (Sonics & Materials; Newtown, CT) at 15% amplitude for 1–2 minutes. Following sonication, samples were heated to 95°C for 5 minutes, and then immediately cooled to room temperature. Protein concentration of each sample was determined using the Pierce BCA Assay Reagent (Thermo Fisher Scientific; Waltham, MA). Protein samples were normalized to the same concentration using 1X SDS-EB, and 10–25 μg total protein was supplemented with 50 mM DTT and 4X LDS sample buffer to a volume of 25 μl. Samples were heated to 70°C for 10 minutes, cooled to room temperature, and separated by SDS-PAGE on 4–12% NuPage gradient gels (Life Technologies) in 1X MOPS buffer for 1–2 hours at 185V. Proteins were transferred overnight onto PVDF membrane (EMD Millipore; Stafford, VA) and subsequently immunoblotted. Primary antibodies used were: rabbit anti-PML (Bethyl Laboratories; Montgomery, TX; dilution 1:1000); rabbit anti-Sp100 (Sigma Corporation; St. Louis, MO; dilution 1:1000–1:2000); mouse anti-α tubulin (Sigma Corporation; St. Louis, MO; dilution 1:10,000); rabbit anti-GFP (AbCam; Cambridge, MA). Species appropriate secondary antibodies conjugated to horseradish peroxidase (Thermo-Pierce; Waltham, MA) were used at 1:10,000 and detected using SuperSignal WestDura Western Detection Reagent (Life Technologies; Forest City, CA). The chemiluminescent signal was collected with a Kodak Bioimager 6000 (Carestream Health; Rochester, NY) or SynGene G:Box (SynGene USA; Frederick, MD) and the resulting signal was quantified.

### Chromatin immunoprecipitation

Chromatin samples from sub-confluent, confluent, and differentiated CIN612-9E cells were prepared as previously described [62]. Optimal conditions for shearing viral chromatin to 200–500 bp fragments were determined by Southern blot analysis to be similar to that of cellular DNA, and subsequently shearing of total DNA was monitored by agarose gel electrophoresis/ethidium bromide staining. For each cell culture condition, 20 μg chromatin was incubated overnight at 4°C with 1 μg ChromPure Rabbit IgG, whole molecule (011-000-003 (Jackson)), 2 μg anti-histone H3 antibody (ab1791 (Abcam)), and 1 μg anti-Sp100 antibody (HPA016707 (Sigma)). Dynabeads with protein G (Invitrogen) were blocked in 1 mg/ml ultrapure BSA (Ambion) and incubated with samples at 4°C to precipitate chromatin immunocomplexes. Dynabeads were subjected to wash steps and DNA was purified using the ChIP DNA Clean and Concentrator kit (Zymo Research). Genomic regions of immunoprecipitated HPV chromatin were quantified with qPCR using an HPV31 plasmid standard curve. For Sp100 protein quantifications following elution of chromatin-immunocomplexes, 5 μg of the 20 μg IP was processed for ChIP-qPCR and 15 μg processed for immunoblot analysis. For immunoblot analysis, chromatin eluate was analyzed by Sp100 immunoblot analysis, as described above. Chemiluminescent signal was collected using a SynGene G:Box (SynGene USA) and the resulting signal quantified using GeneTools image analysis software (SynGene). To calculate the absolute number of Sp100 molecules in each sample, an Sp100 standard curve was generated by in vitro translation of the pCMV-TnT-EE Sp100A plasmid using the TnT Quick Coupled Transcription/Translation System (Promega). Empty pCMV-TnT-EE vector was translated as a negative control. ^35^S-methionine (NEN, 1170Ci/mmol) incorporation was determined by TCA precipitation of translated proteins, allowing calculation of moles of Sp100.

### Image collection, processing, and analysis

All images were collected with a Leica SP5 laser scanning confocal microscope (Leica Microsystems) using a 63X oil immersion objective (NA 1.4). 2D images were collected as a single optical slice for all experiments unless otherwise noted. Images for 3D analysis were collected at either 1 μm or 0.13 μm thickness per slice, as noted in the figure legends. Where noted, images were deconvolved using Huygens Essential (Scientific Volume Imaging B.V., VB Hilversum, Netherlands) using manual background selection and a signal-to-noise ratio of 25:1. Final images were processed using Leica AS AF Lite (Leica Microsystems) or IMARIS (v7.7.1; Bitplane AG; Zurich, Switzerland). Exported images were arranged together as a figure, assembled using Adobe Illustrator (Version CS5; Adobe Systems; San Jose, A) and minimally processed as a group using Adobe Photoshop (Version CS5; Adobe Systems; San Jose, CA).

### Quantitative determination of Sp100 and HPV DNA in tissue

Formalin-fixed, paraffin-embedded tissues from normal and HPV16-infected cervix were deparaffinized, rehydrated, and incubated at 96°C for 30 minutes in epitope-retrieval buffer (25 mM Tris-HCl pH 8.5, 1 mM EDTA, 0.05% SDS) [63]. Sections were blocked with 5% normal donkey serum (NDS) in PBS for one hour in a humidifying chamber at 37°C. Sections were incubated at 37°C with rabbit anti-Sp100 polyclonal antibody (Sigma HPA016707; 1:500) for one hour in 5% NDS/PBS, washed and incubated at 37°C with goat anti-rabbit polyclonal antibody conjugated to rhodamine red-X (Jackson ImmunoResearch) for one hour in 5% NDS/PBS and washed three times with PBS/0.05% Tween 20. Sections were incubated at 37°C with anti-E4 antibody conjugated to Alexa 488 (kindly provided by the Doorbar laboratory; 1:1000) for 1 hour in 5% NDS/PBS, washed three times with PBS/0.05% Tween 20. Antibodies bound to tissue were briefly fixed with 3:1 methanol: acetic acid and subsequently 2% paraformaldehyde/PBS. RNA was removed with 1X RNace-IT cocktail (Stratagene) for one hour at 37°C. Sections were dehydrated with an ethanol series and allowed to dry for one hour. 75 ng HPV16 DNA conjugated to Alexa 647 (FISH Tag DNA Multicolor Kit, Invitrogen) in FISH hybridization buffer (Empire Genomics) with 500 ng human Cot-1 DNA (Invitrogen) was applied to each section and sealed with a coverslip. DNA was denatured for 5 minutes at 75°C and hybridized with probe for 20 hours at 37°C with a Thermobrite StatSpin System (Leica). Slides were washed with 1X phosphate buffered detergent (MP Biomedicals), then with 0.5X SSC with 0.1% SDS at 65°C, and again with 1X phosphate buffered detergent. Sections were incubated with 300 nM DAPI, washed three times with PBS, and mounted with Prolong Gold (Invitrogen). Images were collected with a Leica Sp5 scanning confocal microscope with a 63X objective (NA 1.4). Z stacks of individual nuclei were imaged, deconvolved in Huygens Essential (Scientific Volume Imaging B.V., VB Hilversum, Netherlands) and reconstructed as 3D surfaces in IMARIS (v7.7.1; Bitplane AG; Zurich, Switzerland).

### Ethics statement

Primary human keratinocytes were isolated from anonymized neonatal foreskins provided to the Dermatology Branch at NIH from local hospitals. The NIH Institutional Review Board (IRB) approved this process and issued an NIH Institutional Review Board waiver.

## Supporting information

S1 Fig Identification of Sp100 isoforms in primary keratinocytes. A. Schematic of the Sp100 isoform transcripts. Exons are shown as black rectangles and introns are the connecting black lines. The 3’-UTR region is shown in grey at the end of each transcript. The seed region for the “pan”-Sp100 siRNA used in these experiments is highlighted by the brown bar. The colored arrows indicate the regions amplified by qRT-PCR to specifically detect a particular Sp100 isoform (Sp100A, blue; Sp100B, red; Sp100C, green; Sp100HMG, yellow). B. Schematic representing the four major splice variants of Sp100 and their associated protein domains. C. qRT-PCR was performed on cDNA prepared from primary human keratinocytes maintained in F-medium. Primers were designed to coding regions or 3’-untranslated regions specific for each Sp100 isoform (see A). Data displayed are averages of four different experiments using three HFKs strains. Error bars represent +/- SEM. D. Whole cell protein lysates from HFKs transfected with 400 ng of empty vector or an Sp100 isoform expression vector were collected at 48 hours post DNA transfection. Proteins were separated by SDS-PAGE, transferred to PVDF membranes and immunoblotting was performed using an Sp100-specific antibody that recognizes all Sp100 isoforms. The blot is representative of two independent experiments. The most likely designation of isoforms is indicated.
(PDF)

S2 Fig Interferon induces Sp100 isoform gene expression, protein levels and ND10 bodies. HFKs cultured were in F-medium with or without 25 ng/ml of IFN-α for up to 12 hours and analyzed for expression of Sp100A (A), Sp100B (B), Sp100 (C) or Sp100HMG (D) at the time points indicated. The relative expression of all four isoforms (E) and the fold change in expression level from untreated cells (F) are shown. All results are from independent HFKs strains from two separate experiments. All error bars represent +/- SEM. (G) Whole cell lysates from HFKs cultured in F-medium with 25 ng/ml of IFN-α for 24, 48 or 72 hours were separated by SDS-PAGE and transferred to PVDF membranes. Sp100 species were detected using an Sp100 specific antibody. α-tubulin was used as a loading control. Arrows indicate Sp100 species. The right image is an overexposed section of the image of the exact same gel shown on the left. Numbers to the left of the gel indicate molecular weight markers (kDa). Gel is representative of two independent experiments. (H) HFKs grown on glass coverslips and cultured in F-medium with or without 25 ng/ml of IFN-α were fixed with 4% PFA 48 hours post treatment. Cells were stained with PML (green) or Sp100 (red) specific antibodies. The merged image shows the red and green channels overlaid and are single optical slices.
(PDF)

S3 Fig Western blot analysis of Sp100 proteins in growing, confluent, differentiated and interferon treated 9E cells. A. Western blot analysis of Sp100 proteins in whole cell protein extracts from growing, confluent, and CaCl2 differentiated CIN612-9E cells (as analyzed in Fig 9). Confluent cells were also treated 25 ng/ml IFN-α for twenty-four hours to induce Sp100 expression. The image shown is representative of two independent experiments. These protein samples were extracted in parallel with the experiments shown in Fig 9 and S4 Fig. B. The relative proportion of SP100 isoforms was determined from western blots of Sp100 proteins, as described in A, using Genetools software (Syngene). Two independent replicates were analyzed, as shown.
(PDF)

S4 Fig Interferon induces Sp100 binding to HPV31 chromatin. A. Chromatin immunoprecipitation (ChIP) was performed with samples from growing, confluent and differentiated CIN612-9E cells. ChIP was also performed with samples from confluent CIN612-9E cells treated with 25 ng/ml IFN-α for 24 hours. 20 μg chromatin was immunoprecipitated with antisera to either rabbit IgG, histone H3, or Sp100. Viral DNA was quantified with real-time qPCR using primers targeting major regions of the HPV31 genome (Locations of primers are shown in Fig 9). Binding signals were averaged from three independent experiments, and the amounts shown are from the equivalent of 2 μg input chromatin. Error bars represent +/- SEM. B. Chromatin immunoprecipitation (ChIP) was performed with samples from growing and differentiated (calcium treated) CIN612-9E cells, treated with either control siRNA (siCtrl) or siRNA to Sp100 (siSp100), as described in Fig 6. ChIP was also performed with samples from confluent CIN612-9E cells treated with 25 ng/ml IFN-α for 24 hours. Chromatin was immunoprecipitated with antisera to either rabbit IgG, histone H3, or Sp100. Viral DNA was quantified with real-time qPCR using primers targeting major regions of the HPV31 genome (Locations of primers are shown in Fig 9). Binding signals were averaged from two independent experiments, and the amounts shown are from the equivalent of 2μg input chromatin. Error bars represent +/- SEM. Note that the efficiency of induction of differentiation is somewhat variable (particularly in the presence of siRNA and transfection reagents). C. Western blot analysis for Sp100 in whole cell protein extracts of siRNA treated differentiated cells shown in B. A parallel blot was analyzed for tubulin as a loading control. Rep1 and rep2 are two independent replicates.
(PDF)

S5 Fig Quantification of Sp100 binding to HPV31 genomes in growing and differentiated 9E cells. A. Western blot for Sp100 was performed on chromatin immunoprecipitated samples and normalized to an in vitro translated Sp100 protein standard curve. The number of immunoprecipitated Sp100 molecules was calculated relative to the specific activity of incorporated 35S methionine residues in Sp100A, which was generated by in vitro translation of the pCMV-TnT-EE Sp100A plasmid. Empty pCMV-TnT-EE vector was translated as a negative control. B. The absolute quantity of HPV31 genomes in each sample was measured by ChIP-qPCR analysis of input DNA and immunoprecipitated viral DNA against an HPV31 plasmid DNA standard curve. The changes in the ratio of Sp100 protein to HPV31 DNA molecules in the chromatin samples is shown, relative to growing cells. Data is representative of two independent experiments, and each bar is averaged from the four HPV31 primers shown in Fig 9. Error bars represent +/- SEM.
(PDF)

S6 Fig Sp100 shows increased expression in the suprabasal cells of HPV16-infected cervical tissue. Immunofluorescence and FISH procedure is described in Methods. Tile scans images were collected with a Leica SP5 laser scanning confocal microscope (Leica Microsystems) using a 40X oil-immersion objective (NA 1.25. Background signal was manually subtracted by deconvolution in Huygens Essential (Scientific Volume Imaging B.V., VB Hilversum, Netherlands). Duplicate images were imported into IMARIS (v7.7.1; Bitplane AG; Zurich, Switzerland) to create an artificial Z-stack. Surfaces were manually drawn around each nucleus in the image, rendered in 3D and merged into a single surface using the IMARIS XT tool. The relative signal intensity sum of Sp100 across all nuclei was collectively analyzed using color-coded statistics in IMARIS with identical scales between normal and HPV16-infected tissue. The images at the top are not of equivalent exposure, to see the distribution of Sp100 across the tissue, however the actual quantitation was carried on equivalently collected images.
(PDF)

S1 Table Oligonucleotide and siRNA sequences. Oligonucleotide primers and siRNA sequences.
(PDF)

S2 Table siRNA sequences. (PDF)

## References

[1] Egawa N, Egawa K, Griffin H, Doorbar J. Human Papillomaviruses; Epithelial Tropisms, and the Development of Neoplasia. Viruses. 2015;7(7):3863–90. doi: 10.3390/v7072802 26193301 10.3390/v7072802 PMC4517131

[2] McBride AA. Mechanisms and strategies of papillomavirus replication. Biological chemistry. 2017 doi: 10.1515/hsz-2017-0113 . 28315855 10.1515/hsz-2017-0113

[3] Swindle CS, Zou N, Van Tine BA, Shaw GM, Engler JA, Chow LT. Human papillomavirus DNA replication compartments in a transient DNA replication system. Journal of virology. 1999;73(2):1001–9. 9882301 10.1128/jvi.73.2.1001-1009.1999 PMC103920

[4] Day PM, Baker CC, Lowy DR, Schiller JT. Establishment of papillomavirus infection is enhanced by promyelocytic leukemia protein (PML) expression. Proceedings of the National Academy of Sciences of the United States of America. 2004;101(39):14252–7. doi: 10.1073/pnas.0404229101 15383670 10.1073/pnas.0404229101 PMC521143

[5] Day PM, Roden RB, Lowy DR, Schiller JT. The papillomavirus minor capsid protein, L2, induces localization of the major capsid protein, L1, and the viral transcription/replication protein, E2, to PML oncogenic domains. J Virol. 1998;72(1):142–50. 9420209 10.1128/jvi.72.1.142-150.1998 PMC109358

[6] Florin L, Schafer F, Sotlar K, Streeck RE, Sapp M. Reorganization of nuclear domain 10 induced by papillomavirus capsid protein l2. Virology. 2002;295(1):97–107. Epub 2002/05/30. doi: 10.1006/viro.2002.1360 . 12033769 10.1006/viro.2002.1360

[7] Stepp WH, Meyers JM, McBride AA. Sp100 provides intrinsic immunity against human papillomavirus infection. mBio. 2013;4(6):e00845–13. doi: 10.1128/mBio.00845-13 24194542 10.1128/mBio.00845-13 PMC3892783

[8] Habiger C, Jager G, Walter M, Iftner T, Stubenrauch F. Interferon Kappa Inhibits Human Papillomavirus 31 Transcription by Inducing Sp100 Proteins. J Virol. 2015;90(2):694–704. doi: 10.1128/JVI.02137-15 26491169 10.1128/JVI.02137-15 PMC4702707

[9] Bedell MA, Hudson JB, Golub TR, Turyk ME, Hosken M, Wilbanks GD, et al Amplification of human papillomavirus genomes in vitro is dependent on epithelial differentiation. JVirol. 1991;65:2254–60. 1850010 10.1128/jvi.65.5.2254-2260.1991 PMC240574

[10] Klumpp DJ, Laimins LA. Differentiation-induced changes in promoter usage for transcripts encoding the human papillomavirus type 31 replication protein E1. Virology. 1999;257(1):239–46. doi: 10.1006/viro.1999.9636 . 10208937 10.1006/viro.1999.9636

[11] Johansson C, Schwartz S. Regulation of human papillomavirus gene expression by splicing and polyadenylation. Nature reviews Microbiology. 2013;11(4):239–51. Epub 2013/03/12. doi: 10.1038/nrmicro2984 . 23474685 10.1038/nrmicro2984

[12] Spink KM, Laimins LA. Induction of the human papillomavirus type 31 late promoter requires differentiation but not DNA amplification. J Virol. 2005;79(8):4918–26. Epub 2005/03/30. doi: 10.1128/JVI.79.8.4918-4926.2005 15795277 10.1128/JVI.79.8.4918-4926.2005 PMC1069532

[13] Sakakibara N, Chen D, Jang MK, Kang DW, Luecke HF, Wu SY, et al Brd4 is displaced from HPV replication factories as they expand and amplify viral DNA. PLoS Pathog. 2013;9(11):e1003777 doi: 10.1371/journal.ppat.1003777 24278023 10.1371/journal.ppat.1003777 PMC3836737

[14] De Geest K, Turyk ME, Hosken MI, Hudson JB, Laimins LA, Wilbanks GD. Growth and differentiation of human papillomavirus type 31b positive human cervical cell lines. GynecolOncol. 1993;49(3):303–10. doi: 10.1006/gyno.1993.1131 . 8390960 10.1006/gyno.1993.1131

[15] Stepp WH. The Role of the Nuclear Domain 10 Proteins in the Human Papillomavirus Lifecycle: Georgetown University; 2015.

[16] Kypriotou M, Huber M, Hohl D. The human epidermal differentiation complex: cornified envelope precursors, S100 proteins and the ‘fused genes’ family. Experimental dermatology. 2012;21(9):643–9. doi: 10.1111/j.1600-0625.2012.01472.x 22507538 10.1111/j.1600-0625.2012.01472.x

[17] Ruesch MN, Stubenrauch F, Laimins LA. Activation of papillomavirus late gene transcription and genome amplification upon differentiation in semisolid medium is coincident with expression of involucrin and transglutaminase but not keratin-10. JVirol. 1998;72(6):5016–24. 9573271 10.1128/jvi.72.6.5016-5024.1998 PMC110064

[18] Guldner HH, Szostecki C, Schroder P, Matschl U, Jensen K, Luders C, et al Splice variants of the nuclear dot-associated Sp100 protein contain homologies to HMG-1 and a human nuclear phosphoprotein-box motif. J Cell Sci. 1999;112 (Pt 5):733–47. . 9973607 10.1242/jcs.112.5.733

[19] Sternsdorf T, Jensen K, Reich B, Will H. The nuclear dot protein sp100, characterization of domains necessary for dimerization, subcellular localization, and modification by small ubiquitin-like modifiers. J Biol Chem. 1999;274(18):12555–66. . 10212234 10.1074/jbc.274.18.12555

[20] Seeler JS, Marchio A, Losson R, Desterro JM, Hay RT, Chambon P, et al Common properties of nuclear body protein SP100 and TIF1alpha chromatin factor: role of SUMO modification. Mol Cell Biol. 2001;21(10):3314–24. doi: 10.1128/MCB.21.10.3314-3324.2001 11313457 10.1128/MCB.21.10.3314-3324.2001 PMC100253

[21] Bottomley MJ, Collard MW, Huggenvik JI, Liu Z, Gibson TJ, Sattler M. The SAND domain structure defines a novel DNA-binding fold in transcriptional regulation. Nature structural biology. 2001;8(7):626–33. doi: 10.1038/89675 . 11427895 10.1038/89675

[22] Negorev D, Ishov AM, Maul GG. Evidence for separate ND10-binding and homo-oligomerization domains of Sp100. J Cell Sci. 2001;114(Pt 1):59–68. . 11112690 10.1242/jcs.114.1.59

[23] Cuchet-Lourenco D, Boutell C, Lukashchuk V, Grant K, Sykes A, Murray J, et al SUMO pathway dependent recruitment of cellular repressors to herpes simplex virus type 1 genomes. PLoS Pathog. 2011;7(7):e1002123 Epub 2011/07/23. doi: 10.1371/journal.ppat.1002123 21779164 10.1371/journal.ppat.1002123 PMC3136452

[24] Guldner HH, Szostecki C, Grotzinger T, Will H. IFN enhance expression of Sp100, an autoantigen in primary biliary cirrhosis. Journal of immunology. 1992;149(12):4067–73. . 1281200

[25] Negorev DG, Vladimirova OV, Ivanov A, Rauscher F 3rd, Maul GG. Differential role of Sp100 isoforms in interferon-mediated repression of herpes simplex virus type 1 immediate-early protein expression. J Virol. 2006;80(16):8019–29. Epub 2006/07/29. doi: 10.1128/JVI.02164-05 16873258 10.1128/JVI.02164-05 PMC1563809

[26] Negorev DG, Vladimirova OV, Maul GG. Differential functions of interferon-upregulated Sp100 isoforms: herpes simplex virus type 1 promoter-based immediate-early gene suppression and PML protection from ICP0-mediated degradation. J Virol. 2009;83(10):5168–80. Epub 2009/03/13. doi: 10.1128/JVI.02083-08 19279115 10.1128/JVI.02083-08 PMC2682089

[27] Berscheminski J, Wimmer P, Brun J, Ip WH, Groitl P, Horlacher T, et al Sp100 isoform-specific regulation of human adenovirus 5 gene expression. J Virol. 2014;88(11):6076–92. Epub 2014/03/14. doi: 10.1128/JVI.00469-14 24623443 10.1128/JVI.00469-14 PMC4093896

[28] Sakakibara N, Chen D, McBride AA. Papillomaviruses use recombination-dependent replication to vegetatively amplify their genomes in differentiated cells. PLoS Pathog. 2013;9(7):e1003321 doi: 10.1371/journal.ppat.1003321 23853576 10.1371/journal.ppat.1003321 PMC3701714

[29] Nakahara T, Lambert PF. Induction of promyelocytic leukemia (PML) oncogenic domains (PODs) by papillomavirus. Virology. 2007;366(2):316–29. doi: 10.1016/j.virol.2007.04.032 17543368 10.1016/j.virol.2007.04.032 PMC2777652

[30] Tavalai N, Adler M, Scherer M, Riedl Y, Stamminger T. Evidence for a dual antiviral role of the major nuclear domain 10 component Sp100 during the immediate-early and late phases of the human cytomegalovirus replication cycle. J Virol. 2011;85(18):9447–58. Epub 2011/07/08. doi: 10.1128/JVI.00870-11 21734036 10.1128/JVI.00870-11 PMC3165758

[31] Adler M, Tavalai N, Muller R, Stamminger T. Human cytomegalovirus immediate-early gene expression is restricted by the nuclear domain 10 component Sp100. J Gen Virol. 2011;92(Pt 7):1532–8. doi: 10.1099/vir.0.030981-0 . 21471311 10.1099/vir.0.030981-0

[32] Walters MS, Kyratsous CA, Silverstein SJ. The RING finger domain of Varicella-Zoster virus ORF61p has E3 ubiquitin ligase activity that is essential for efficient autoubiquitination and dispersion of Sp100-containing nuclear bodies. J Virol. 2010;84(13):6861–5. doi: 10.1128/JVI.00335-10 20392849 10.1128/JVI.00335-10 PMC2903287

[33] Neumann F, Czech-Sioli M, Schmidt C, Dobner T, Grundhoff A, Schreiner S, et al Replication of merkel cell polyomavirus induces reorganization of promyelocytic leukemia nuclear bodies. J Gen Virol. 2016 Epub 2016/09/02. doi: 10.1099/jgv.0.000593 . 27580912 10.1099/jgv.0.000593

[34] Reuter N, Schilling EM, Scherer M, Muller R, Stamminger T. The ND10 component promyelocytic leukemia protein acts as an E3 ligase for SUMOylation of the major immediate-early protein IE1 of human cytomegalovirus. J Virol. 2017 Epub 2017/03/03. doi: 10.1128/jvi.02335-16 . 28250117 10.1128/JVI.02335-16 PMC5411614

[35] Full F, Reuter N, Zielke K, Stamminger T, Ensser A. Herpesvirus saimiri antagonizes nuclear domain 10-instituted intrinsic immunity via an ORF3-mediated selective degradation of cellular protein Sp100. J Virol. 2012;86(7):3541–53. Epub 2012/01/27. doi: 10.1128/JVI.06992-11 22278248 10.1128/JVI.06992-11 PMC3302493

[36] Bell P, Lieberman PM, Maul GG. Lytic but not latent replication of epstein-barr virus is associated with PML and induces sequential release of nuclear domain 10 proteins. JVirol. 2000;74(24):11800–10. 11090180 10.1128/jvi.74.24.11800-11810.2000 PMC112463

[37] Xu P, Mallon S, Roizman B. PML plays both inimical and beneficial roles in HSV-1 replication. Proceedings of the National Academy of Sciences of the United States of America. 2016;113(21):E3022–E8. doi: 10.1073/pnas.1605513113 27162364 10.1073/pnas.1605513113 PMC4889406

[38] Scherer M, Stamminger T. Emerging Role of PML Nuclear Bodies in Innate Immune Signaling. J Virol. 2016;90(13):5850–4. doi: 10.1128/JVI.01979-15 27053550 10.1128/JVI.01979-15 PMC4907236

[39] Xu P, Roizman B. The SP100 component of ND10 enhances accumulation of PML and suppresses replication and the assembly of HSV replication compartments. Proc Natl Acad Sci U S A. 2017 doi: 10.1073/pnas.1703395114 . 28439026 10.1073/pnas.1703395114 PMC5441741

[40] McKinney CC, Hussmann KL, McBride AA. The Role of the DNA Damage Response throughout the Papillomavirus Life Cycle. Viruses. 2015;7(5):2450–69. Epub 2015/05/27. doi: 10.3390/v7052450 26008695 10.3390/v7052450 PMC4452914

[41] Carbone R, Pearson M, Minucci S, Pelicci PG. PML NBs associate with the hMre11 complex and p53 at sites of irradiation induced DNA damage. Oncogene. 2002;21(11):1633–40. Epub 2002/03/16. doi: 10.1038/sj.onc.1205227 11896594 10.1038/sj.onc.1205227

[42] Ishov AM, Sotnikov AG, Negorev D, Vladimirova OV, Neff N, Kamitani T, et al PML is critical for ND10 formation and recruits the PML-interacting protein daxx to this nuclear structure when modified by SUMO-1. J Cell Biol. 1999;147(2):221–34. 10525530 10.1083/jcb.147.2.221 PMC2174231

[43] Bischof O, Kim SH, Irving J, Beresten S, Ellis NA, Campisi J. Regulation and localization of the Bloom syndrome protein in response to DNA damage. J Cell Biol. 2001;153(2):367–80. Epub 2001/04/20. 11309417 10.1083/jcb.153.2.367 PMC2169463

[44] Anacker DC, Gautam D, Gillespie KA, Chappell WH, Moody CA. Productive replication of human papillomavirus 31 requires DNA repair factor Nbs1. J Virol. 2014;88(15):8528–44. doi: 10.1128/JVI.00517-14 24850735 10.1128/JVI.00517-14 PMC4135936

[45] Moody CA, Laimins LA. Human papillomaviruses activate the ATM DNA damage pathway for viral genome amplification upon differentiation. PLoS Pathog. 2009;5(10):e1000605 doi: 10.1371/journal.ppat.1000605 19798429 10.1371/journal.ppat.1000605 PMC2745661

[46] Nakahara T, Peh WL, Doorbar J, Lee D, Lambert PF. Human papillomavirus type 16 E1^E4 contributes to multiple facets of the papillomavirus life cycle. JVirol. 2005;79(20):13150–65. 16189016 10.1128/JVI.79.20.13150-13165.2005 PMC1235822

[47] Chang YE, Laimins LA. Microarray analysis identifies interferon-inducible genes and Stat-1 as major transcriptional targets of human papillomavirus type 31. JVirol. 2000;74(9):4174–82. 10756030 10.1128/jvi.74.9.4174-4182.2000 PMC111932

[48] Newhart A, Negorev DG, Rafalska-Metcalf IU, Yang T, Maul GG, Janicki SM. Sp100A promotes chromatin decondensation at a cytomegalovirus-promoter-regulated transcription site. Mol Biol Cell. 2013;24(9):1454–68. Epub 2013/03/15. doi: 10.1091/mbc.E12-09-0669 23485562 10.1091/mbc.E12-09-0669 PMC3639056

[49] Isaac A, Wilcox KW, Taylor JL. SP100B, a repressor of gene expression preferentially binds to DNA with unmethylated CpGs. J Cell Biochem. 2006;98(5):1106–22. doi: 10.1002/jcb.20841 . 16775843 10.1002/jcb.20841

[50] Wilcox KW, Sheriff S, Isaac A, Taylor JL. SP100B is a repressor of gene expression. J Cell Biochem. 2005;95(2):352–65. Epub 2005/03/22. doi: 10.1002/jcb.20434 . 15778999 10.1002/jcb.20434

[51] Zhang XJ, Zhao D, Xiong XZ, He ZM, Li HT. Multifaceted Histone H3 Methylation and Phosphorylation Readout by the Plant Homeodomain Finger of Human Nuclear Antigen Sp100C. Journal of Biological Chemistry. 2016;291(24):12786–98. doi: 10.1074/jbc.M116.721159 27129259 10.1074/jbc.M116.721159 PMC4933467

[52] Goldsborough MD, DiSilvestre D, Temple GF, Lorincz AT. Nucleotide sequence of human papillomavirus type 31: a cervical neoplasia-associated virus. Virology. 1989;171(1):306–11. . 2545036 10.1016/0042-6822(89)90545-x

[53] Flores ER, Lambert PF. Evidence for a switch in the mode of human papillomavirus type 16 DNA replication during the viral life cycle. JVirol. 1997;71(10):7167–79. 9311789 10.1128/jvi.71.10.7167-7179.1997 PMC192056

[54] Boshart M, Gissmann L, Ikenberg H, Kleinheinz A, Scheurlen W, zur Hausen H. A new type of papillomavirus DNA, its presence in genital cancer biopsies and in cell lines derived from cervical cancer. EMBO J. 1984;3(5):1151–7. 6329740 10.1002/j.1460-2075.1984.tb01944.x PMC557488

[55] Banerjee NS, Wang HK, Broker TR, Chow LT. Human papillomavirus (HPV) E7 induces prolonged G2 following S phase reentry in differentiated human keratinocytes. J Biol Chem. 2011;286(17):15473–82. Epub 2011/02/16. doi: 10.1074/jbc.M110.197574 21321122 10.1074/jbc.M110.197574 PMC3083224

[56] Sakakibara N, Mitra R, McBride AA. The papillomavirus E1 helicase activates a cellular DNA damage response in viral replication foci. J Virol. 2011;85(17):8981–95. doi: 10.1128/JVI.00541-11 21734054 10.1128/JVI.00541-11 PMC3165833

[57] Del Vecchio AM, Romanczuk H, Howley PM, Baker CC. Transient replication of human papillomavirus DNAs. JVirol. 1992;66:5949–58. 1326651 10.1128/jvi.66.10.5949-5958.1992 PMC241472

[58] Chapman S, Liu X, Meyers C, Schlegel R, McBride AA. Human keratinocytes are efficiently immortalized by a Rho kinase inhibitor. The Journal of clinical investigation. 2010;120(7):2619–26. doi: 10.1172/JCI42297 20516646 10.1172/JCI42297 PMC2898606

[59] Rheinwald JG, Green H. Serial cultivation of strains of human epidermal keratinocytes: the formation of keratinizing colonies from single cells. Cell. 1975;6(3):331–43. 1052771 10.1016/s0092-8674(75)80001-8

[60] Gorman CM, Howard BH, Reeves R. Expression of recombinant plasmids in mammalian cells is enhanced by sodium butyrate. Nucleic Acids Research. 1983;11:7631–48. 6316266 10.1093/nar/11.21.7631 PMC326508

[61] Rodriguez AJ, Shenoy SM, Singer RH, Condeelis J. Visualization of mRNA translation in living cells. J Cell Biol. 2006;175(1):67–76. doi: 10.1083/jcb.200512137 17030983 10.1083/jcb.200512137 PMC2064499

[62] Dooley KE, Warburton A, McBride AA. Tandemly Integrated HPV16 Can Form a Brd4-Dependent Super-Enhancer-Like Element That Drives Transcription of Viral Oncogenes. mBio. 2016;7(5):e01446–16. Epub 2016/09/15. doi: 10.1128/mBio.01446-16 27624132 10.1128/mBio.01446-16 PMC5021809

[63] Syrbu SI, Cohen MB. An enhanced antigen-retrieval protocol for immunohistochemical staining of formalin-fixed, paraffin-embedded tissues. Methods in molecular biology. 2011;717:101–10. doi: 10.1007/978-1-61779-024-9_6 . 21370027 10.1007/978-1-61779-024-9_6

