# Supplementary figures and images for: Sp100 colocalizes with HPV replication foci and restricts the productive stage of the infectious cycle
Source: PLoS Pathog. 2017 Oct 2;13(10):e1006660. doi: 10.1371/journal.ppat.1006660 (PMC5638619; doi:10.1371/journal.ppat.1006660)

**A**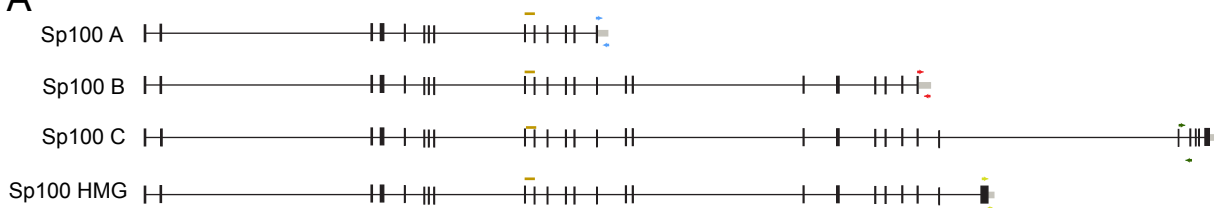**B**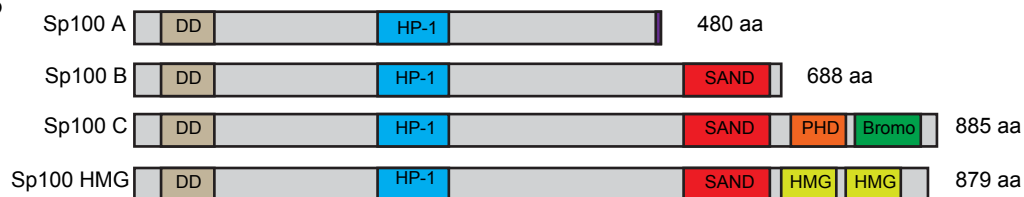**C**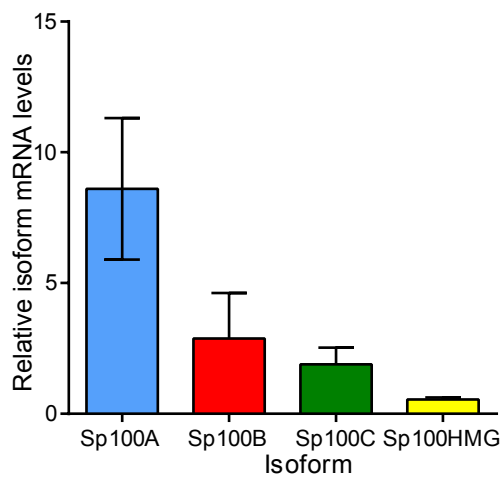**D**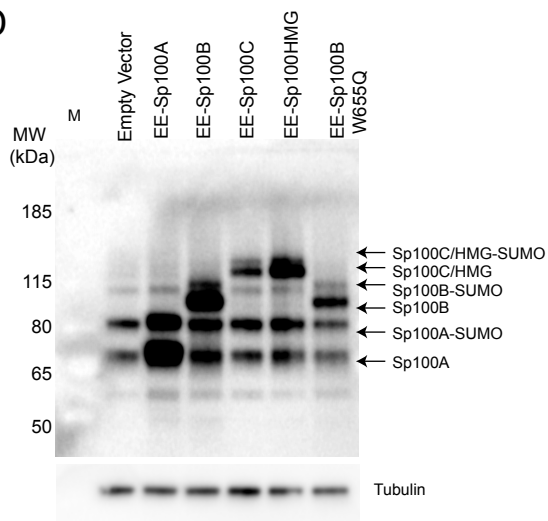

Supplement: S1 Fig — A. Schematic of the Sp100 isoform transcripts. Exons are shown as black rectangles and introns are the connecting black lines. The 3’-UTR region is shown in grey at the end of each transcript. The seed region for the “pan”-Sp100 siRNA used in these experiments is highlighted by the brown bar. The colored arrows indicate the regions amplified by qRT-PCR to specifically detect a particular Sp100 isoform (Sp100A, blue; Sp100B, red; Sp100C, green; Sp100HMG, yellow). B. Schematic representing the four major splice variants of Sp100 and their associated protein domains. C. qRT-PCR was performed on cDNA prepared from primary human keratinocytes maintained in F-medium. Primers were designed to coding regions or 3’-untranslated regions specific for each Sp100 isoform (see A). Data displayed are averages of four different experiments using three HFKs strains. Error bars represent +/- SEM. D. Whole cell protein lysates from HFKs transfected with 400 ng of empty vector or an Sp100 isoform expression vector were collected at 48 hours post DNA transfection. Proteins were separated by SDS-PAGE, transferred to PVDF membranes and immunoblotting was performed using an Sp100-specific antibody that recognizes all Sp100 isoforms. The blot is representative of two independent experiments. The most likely designation of isoforms is indicated. (PDF) [file ppat.1006660.s001.pdf]

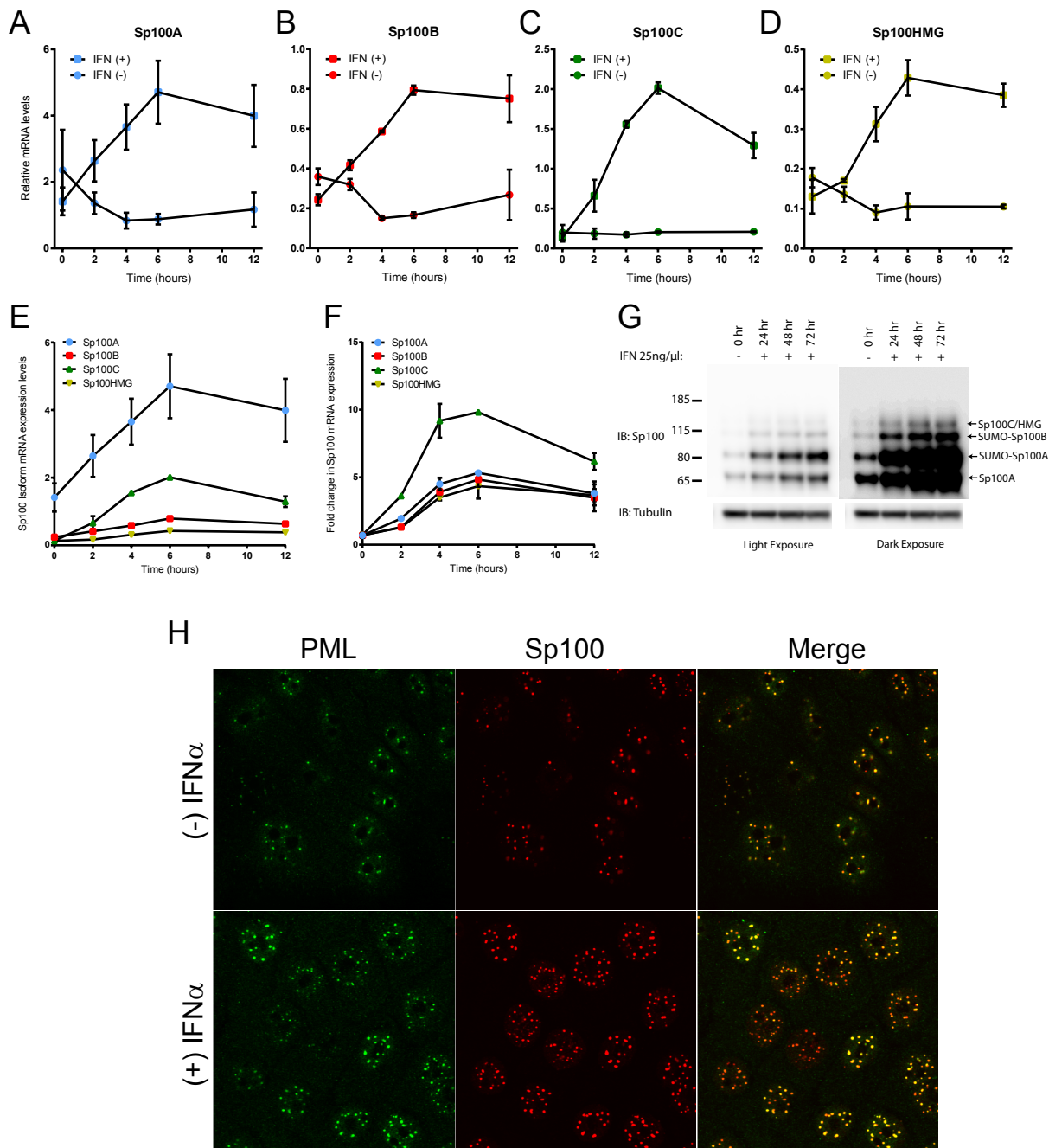

Supplement: S2 Fig — HFKs cultured were in F-medium with or without 25 ng/ml of IFN-α for up to 12 hours and analyzed for expression of Sp100A (A), Sp100B (B), Sp100 (C) or Sp100HMG (D) at the time points indicated. The relative expression of all four isoforms (E) and the fold change in expression level from untreated cells (F) are shown. All results are from independent HFKs strains from two separate experiments. All error bars represent +/- SEM. (G) Whole cell lysates from HFKs cultured in F-medium with 25 ng/ml of IFN-α for 24, 48 or 72 hours were separated by SDS-PAGE and transferred to PVDF membranes. Sp100 species were detected using an Sp100 specific antibody. α-tubulin was used as a loading control. Arrows indicate Sp100 species. The right image is an overexposed section of the image of the exact same gel shown on the left. Numbers to the left of the gel indicate molecular weight markers (kDa). Gel is representative of two independent experiments. (H) HFKs grown on glass coverslips and cultured in F-medium with or without 25 ng/ml of IFN-α were fixed with 4% PFA 48 hours post treatment. Cells were stained with PML (green) or Sp100 (red) specific antibodies. The merged image shows the red and green channels overlaid and are single optical slices. (PDF) [file ppat.1006660.s002.pdf]

A.

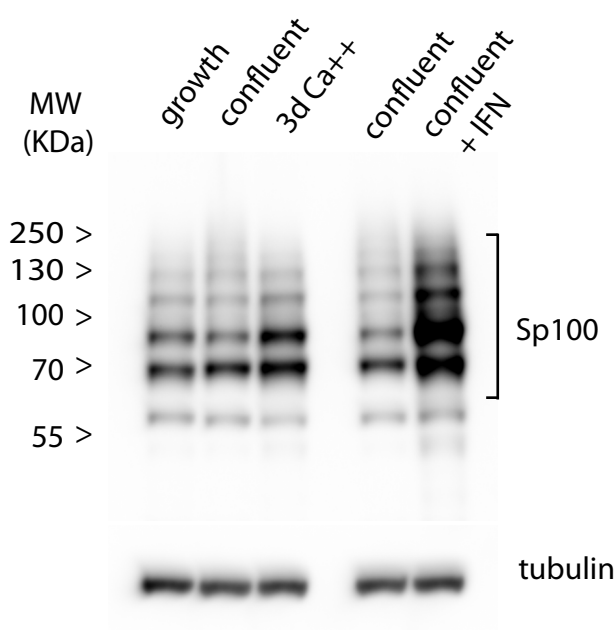

B.

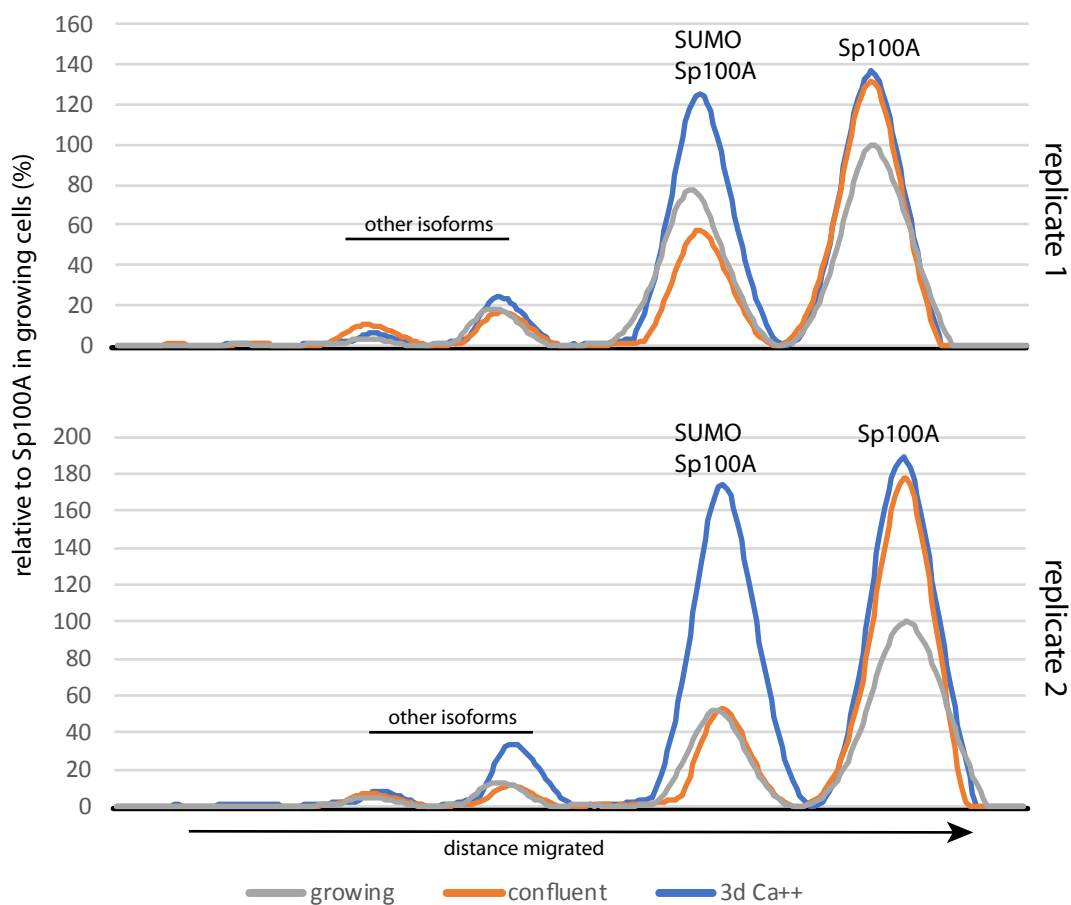

Supplement: S3 Fig — A. Western blot analysis of Sp100 proteins in whole cell protein extracts from growing, confluent, and CaCl2 differentiated CIN612-9E cells (as analyzed in Fig 9). Confluent cells were also treated 25 ng/ml IFN-α for twenty-four hours to induce Sp100 expression. The image shown is representative of two independent experiments. These protein samples were extracted in parallel with the experiments shown in Fig 9 and S4 Fig. B. The relative proportion of SP100 isoforms was determined from western blots of Sp100 proteins, as described in A, using Genetools software (Syngene). Two independent replicates were analyzed, as shown. (PDF) [file ppat.1006660.s003.pdf]

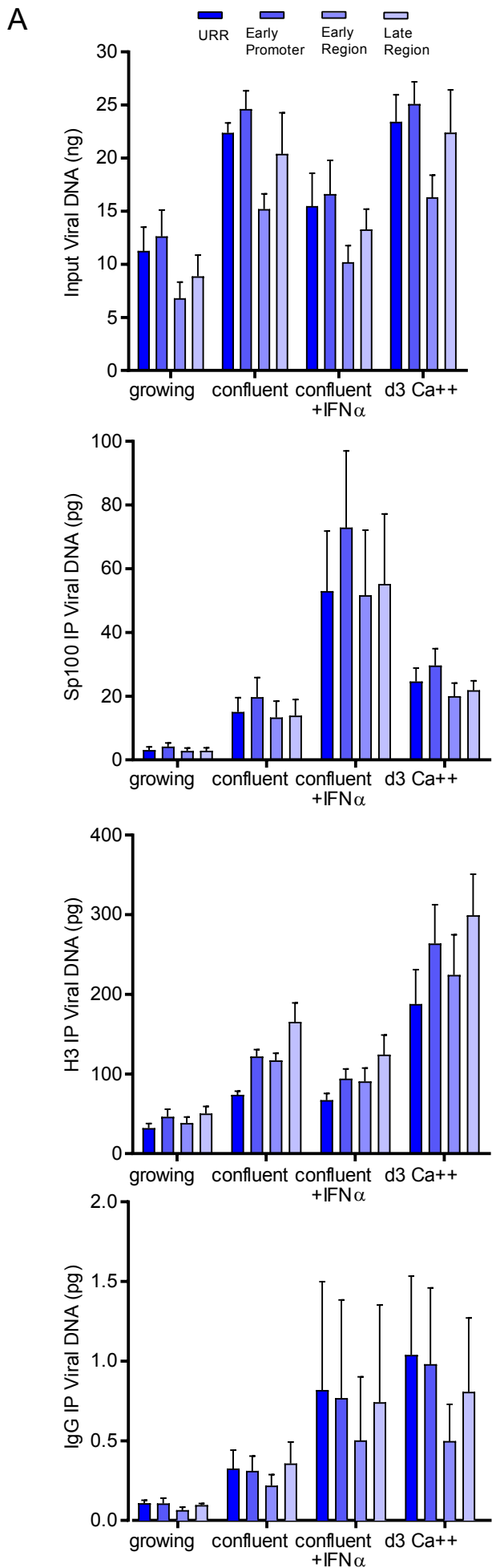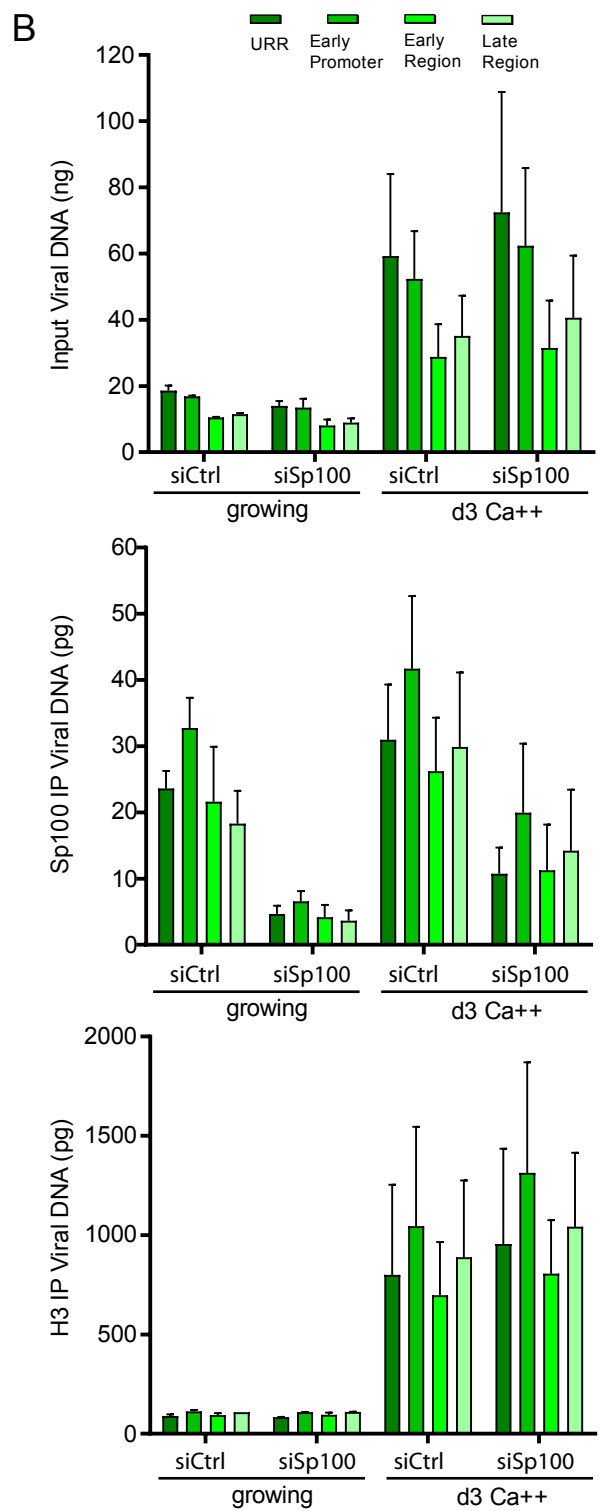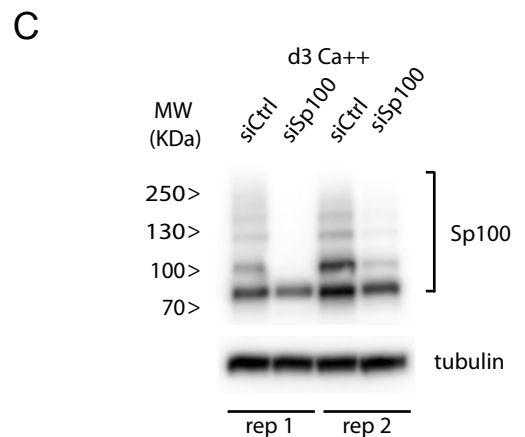

Supplement: S4 Fig — A. Chromatin immunoprecipitation (ChIP) was performed with samples from growing, confluent and differentiated CIN612-9E cells. ChIP was also performed with samples from confluent CIN612-9E cells treated with 25 ng/ml IFN-α for 24 hours. 20 μg chromatin was immunoprecipitated with antisera to either rabbit IgG, histone H3, or Sp100. Viral DNA was quantified with real-time qPCR using primers targeting major regions of the HPV31 genome (Locations of primers are shown in Fig 9). Binding signals were averaged from three independent experiments, and the amounts shown are from the equivalent of 2 μg input chromatin. Error bars represent +/- SEM. B. Chromatin immunoprecipitation (ChIP) was performed with samples from growing and differentiated (calcium treated) CIN612-9E cells, treated with either control siRNA (siCtrl) or siRNA to Sp100 (siSp100), as described in Fig 6. ChIP was also performed with samples from confluent CIN612-9E cells treated with 25 ng/ml IFN-α for 24 hours. Chromatin was immunoprecipitated with antisera to either rabbit IgG, histone H3, or Sp100. Viral DNA was quantified with real-time qPCR using primers targeting major regions of the HPV31 genome (Locations of primers are shown in Fig 9). Binding signals were averaged from two independent experiments, and the amounts shown are from the equivalent of 2μg input chromatin. Error bars represent +/- SEM. Note that the efficiency of induction of differentiation is somewhat variable (particularly in the presence of siRNA and transfection reagents). C. Western blot analysis for Sp100 in whole cell protein extracts of siRNA treated differentiated cells shown in B. A parallel blot was analyzed for tubulin as a loading control. Rep1 and rep2 are two independent replicates. (PDF) [file ppat.1006660.s004.pdf]

A

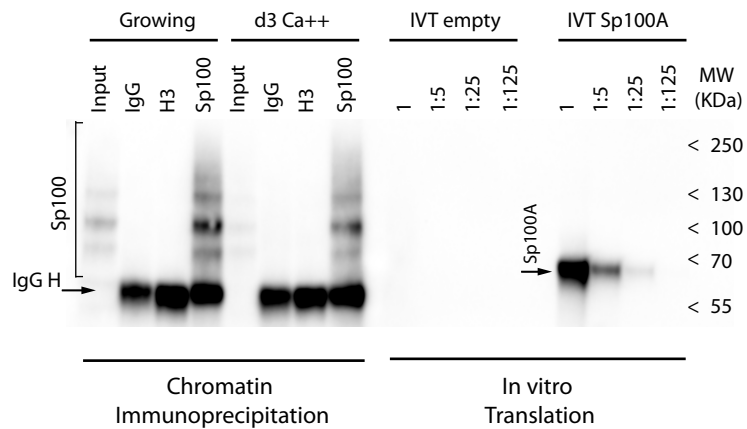

B

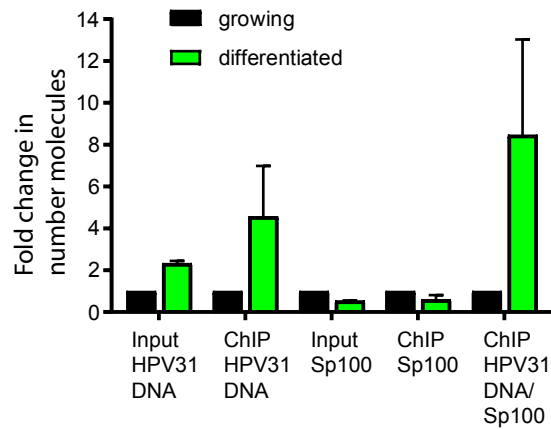

Supplement: S5 Fig — A. Western blot for Sp100 was performed on chromatin immunoprecipitated samples and normalized to an in vitro translated Sp100 protein standard curve. The number of immunoprecipitated Sp100 molecules was calculated relative to the specific activity of incorporated 35S methionine residues in Sp100A, which was generated by in vitro translation of the pCMV-TnT-EE Sp100A plasmid. Empty pCMV-TnT-EE vector was translated as a negative control. B. The absolute quantity of HPV31 genomes in each sample was measured by ChIP-qPCR analysis of input DNA and immunoprecipitated viral DNA against an HPV31 plasmid DNA standard curve. The changes in the ratio of Sp100 protein to HPV31 DNA molecules in the chromatin samples is shown, relative to growing cells. Data is representative of two independent experiments, and each bar is averaged from the four HPV31 primers shown in Fig 9. Error bars represent +/- SEM. (PDF) [file ppat.1006660.s005.pdf]
