# Supplementary material for: Sp100 colocalizes with HPV replication foci and restricts the productive stage of the infectious cycle
Source: PLoS Pathog. 2017 Oct 2;13(10):e1006660. doi: 10.1371/journal.ppat.1006660 (PMC5638619; doi:10.1371/journal.ppat.1006660)
Supplement: S6 Fig — Immunofluorescence and FISH procedure is described in Methods. Tile scans images were collected with a Leica SP5 laser scanning confocal microscope (Leica Microsystems) using a 40X oil-immersion objective (NA 1.25. Background signal was manually subtracted by deconvolution in Huygens Essential (Scientific Volume Imaging B.V., VB Hilversum, Netherlands). Duplicate images were imported into IMARIS (v7.7.1; Bitplane AG; Zurich, Switzerland) to create an artificial Z-stack. Surfaces were manually drawn around each nucleus in the image, rendered in 3D and merged into a single surface using the IMARIS XT tool. The relative signal intensity sum of Sp100 across all nuclei was collectively analyzed using color-coded statistics in IMARIS with identical scales between normal and HPV16-infected tissue. The images at the top are not of equivalent exposure, to see the distribution of Sp100 across the tissue, however the actual quantitation was carried on equivalently collected images. (PDF) [file ppat.1006660.s006.pdf]

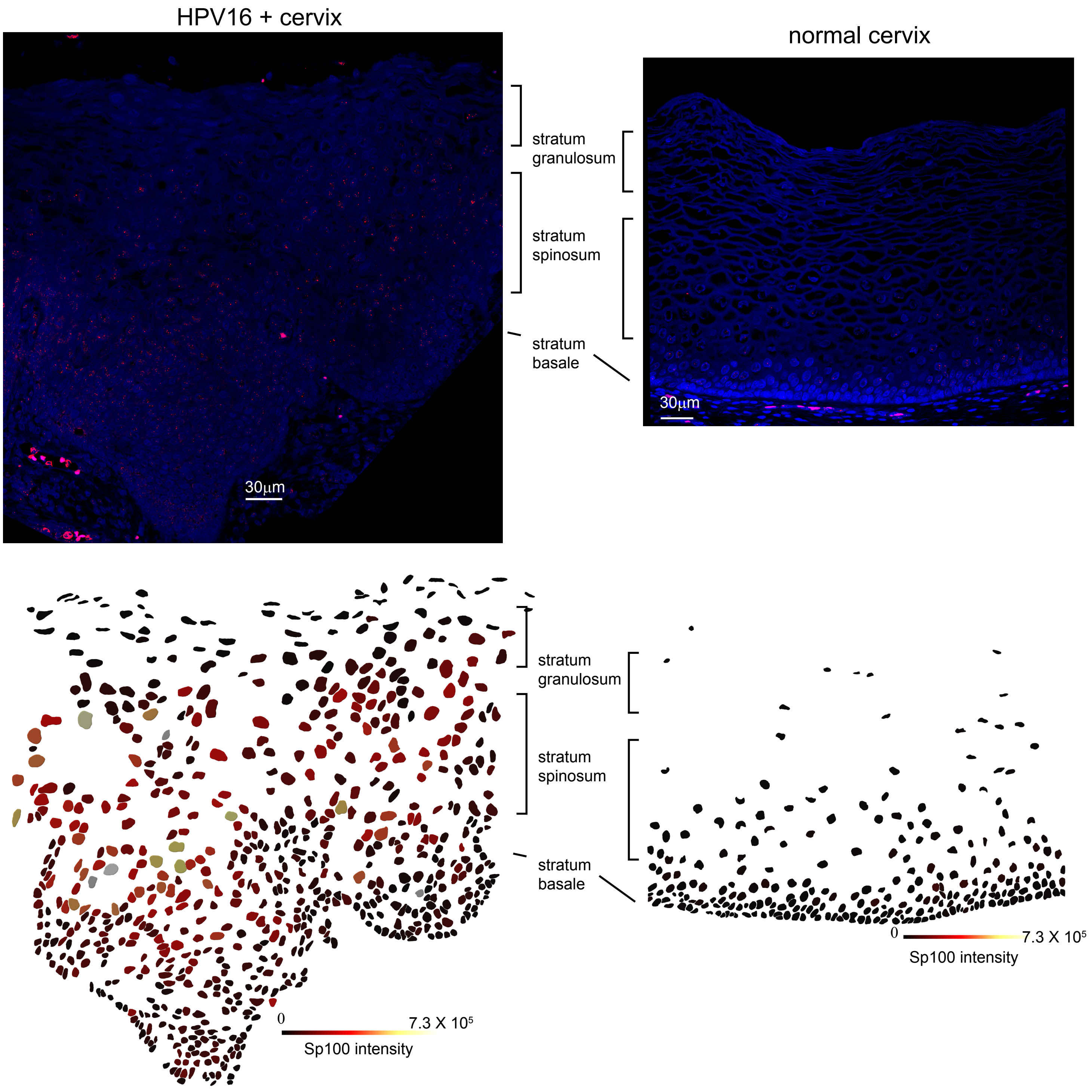

HPV16 + cervix
normal cervix
stratum granulosum
stratum spinosum
stratum basale
30μm
30μm
stratum granulosum
stratum spinosum
stratum basale
0
7.3 X 10⁵
Sp100 intensity
0
7.3 X 10⁵
Sp100 intensity
