# Supplementary material for: Sp100 colocalizes with HPV replication foci and restricts the productive stage of the infectious cycle
Source: PLoS Pathog. 2017 Oct 2;13(10):e1006660. doi: 10.1371/journal.ppat.1006660 (PMC5638619; doi:10.1371/journal.ppat.1006660)
Supplement: S1 Table — Oligonucleotide primers and siRNA sequences. (PDF) [file ppat.1006660.s007.pdf]

Supplementary Table 1 Oligonucleotide sequences

Gene (Accession #)	Nucleotide numbers	Primer Use (strand)	Sequence (5'-3')
GAPDH (NM_001256799.1)	346-365	qRT-PCR (sense)	5'-GATTCCACCCATGGCAAATT-3'
GAPDH (NM_001256799.1)	416-394	qRT-PCR (antisense)	5'-GGATTTCCATTGATGACAAGCTT-3'
TBP (NM_001172085.1)	605-629	qRT-PCR (sense)	5'-TAAACTTGACCTAAAGACCATTGCA-3'
TBP (NM_001172085.1)	672-653	qRT-PCR (antisense)	5'-CAGCAAACCGCTTGGGATTA-3'
beta-Actin (NG_007992.1)	7831-7848	DNA qPCR beta-actin sense)	5'-ATCGTCCACCGCAAATGC-3'
beta-Actin (NG_007992.1)	7909-7884	DNA qPCR beta-actin (antisense)	5'-CGCAAGTTAGGTTTTGTCAAGAAA-3'
Beta-actin (NG_007992.1)	7832-7848	DNA qPCR (sense)	5'-TCGTCCACCGCAAATGC-3'
Beta-actin (NG_007992.1)	7907-7884	DNA qPCR (antisense)	5'-CGCAAGTTAGGTTTTGTCAAGAAA-3'
HPV31 E6*I	186-210, 413-416	qRT-PCR (sense)	5'-AGATTGAATTGTGTCTACTGCAAAGGTGT-3'
HPV31 E6*I	520-498	qRT-PCR (antisense)	5'-GCTATGCAACGTCCTGTCCACCT-3'
HPV31 E1^E4	857-877, 3294-3296	qRT-PCR (sense)	5'-CTACAATGGCTGATCCAGCAGCA-3'
HPV31 E1^E4	3405-3387	qRT-PCR (antisense)	5'-CGCCGCACACCTTCACTGG-3'
HPV31 E2 877^2646	862-877, 2646-2648	qRT-PCR (sense)	5'-ATGGCTGATCCAGCAGGAC-3'
HPV31 E2 877^2646	2712-2691	qRT-PCR (antisense)	5'-CGTTGAGAAAGAGTCTCCATCG-3'
HPV31 L1 3590^5552	3562-3590, 5552-5554	qRT-PCR (sense)	5'-TGCAACTACACCTATAATACACTTAAAAGATG-3'
HPV31 L1 3590^5552	5641-5619	qRT-PCR (antisense)	5'-TCGTGTTACATATTCATCCGTGC-3'
HPV31 DNA	618-640	DNA qPCR (sense)	5'-CTGACCTCCACTGTTATGAGCAA-3'
HPV31 DNA	686-663	DNA qPCR (antisense)	5'-CAGCTGGACTGTCTATGACATCCT-3'
SP100, Isoform A (NM_001206702.1)	1594-1613	qRT-PCR (sense)	5'-ACTTGGCCTGCAGAATGTCA-3'
SP100, Isoform A (NM_001206702.1)	1676-1655	qRT-PCR (antisense)	5'-CAAGGTAGTGAAGGTGCTCAGA-3'
SP100, Isoform B (NM_001206701.1)	2212-2236	qRT-PCR (sense)	5'-TCTGCCAATGTCTCGTCTATTATGT-3'
SP100, Isoform B (NM_001206701.1)	2291-2263	qRT-PCR (antisense)	5'-TTATGATGATGGGTCAATTTAAAGACTGT-3'

SP100, Isoform C (NM_001080391.1)	2455-2474	qRT-PCR (sense)	5’-CTGCCTGAGGAGCAGTTGAA-3’
SP100, Isoform C (NM_001080391.1)	2538-2519	qRT-PCR (antisense)	5’-CGGTTCTGAGGCGAAAAAGC-3’
SP100, Isoform HMG (NM_003113.3)	2224-2244	qRT-PCR (sense)	5’-GTTGACCCTTGTGAGGAGCAT-3’
SP100, Isoform HMG (NM_003113.3)	2365-2345	qRT-PCR (antisense)	5’-TGTCCGCCTTTGCCATATCTT-3’
URR	7527-7548	ChIP-qPCR (sense)	5’-AGTAGTTCTGCGGTTTTTGGTT-3’
URR	7615-7594	ChIP-qPCR (antisense)	5’-TATGTTGGCAAGGTGTGTTAGG-3’
Early promoter	43-62	ChIP-qPCR (sense)	5’-AAGTGGTGAACCGAAAACGG-3’
Early promoter	114-95	ChIP-qPCR (antisense)	5’-TGAACATGGCGTCTGTAGGT-3’
Early region	3410-3429	ChIP-qPCR (sense)	5’-ACGACGTCTACTAAGCGACC-3’
Early region	3550-3531	ChIP-qPCR (antisense)	5’-AGCCCTTGTTTGGTTTGTGC-3’
Late Region	5676-5695	ChIP-qPCR (sense)	5’-TGCTTACAGTAGGCCATCCA-3’
Late Region	5794-5774	ChIP-qPCR (antisense)	5’-TGGATCTGGTAAACGAACCCT-3’
