# Supplementary material for: Sp100 colocalizes with HPV replication foci and restricts the productive stage of the infectious cycle
Source: PLoS Pathog. 2017 Oct 2;13(10):e1006660. doi: 10.1371/journal.ppat.1006660 (PMC5638619; doi:10.1371/journal.ppat.1006660)
Supplement: S2 Table — (PDF) [file ppat.1006660.s008.pdf]

Supplementary Table 2. siRNA sequences

**Target mRNA**	**Sequence (5’-3’)**
Non-targeting Control	5’-UGGUUUACAUGUCGACUAA-3’
	5’-UGGUUUACAUGUUGUGUGA-3’
	5’-UGGUUUACAUGUUUUCUGA-3’
	5’-UGGUUUACAUGUUUUCCUA-3’
PML	5’-GCAACCAGUCGGUGCGUGA-3’
	5’-GGACAUGCACGGUUUCCUG-3’
	5’-GAGCUCAAGUGCGACAUCA-3’
	5’-GGAAAGAUGCAGCUGUAUC-3’
SP100	5’-GAAGUGAGCCUGUGAUCAA-3’
	5’-AGGCAUAGAUCUAAAGUAA-3’
	5’-UACCAGAGCCCAUGGAUUU-3’
	5’-GAAGGGCACUCUAUAUAAG-3’
